# Comparative *Penicillium* spp. Transcriptomics: Conserved Pathways and Processes Revealed in Ungerminated Conidia and during Postharvest Apple Fruit Decay

**DOI:** 10.3390/microorganisms10122414

**Published:** 2022-12-06

**Authors:** Holly P. Bartholomew, Franz J. Lichtner, Michael Bradshaw, Verneta L. Gaskins, Jorge M. Fonseca, Joan W. Bennett, Wayne M. Jurick

**Affiliations:** 1 Food Quality Laboratory, Beltsville Agricultural Research Center, USDA-ARS, Beltsville, MD 20705, USA; 2 U.S. Army Corps of Engineers Engineer Research & Development Center, Cold Regions Research & Engineering Laboratory, 72 Lyme Road, Hanover, NH 03755, USA; 3 Department of Organismic and Evolutionary Biology, Harvard University, Cambridge, MA 02138, USA; 4 Department of Plant Biology, Rutgers, The State University of New Jersey, New Brunswick, NJ 08901, USA

**Keywords:** apple fruit, blue mold, *Penicillium* spp., fungal phytopathogens, postharvest decay, transcriptomics

## Abstract

Blue mold, caused by *Penicillium* spp., is an impactful postharvest disease resulting in significant economic losses due to reduced pome fruit quality and mycotoxin contamination. Using two *Penicillium* species with different levels of aggressiveness, transcriptomics were implemented in order to identify genes expressed during apple fruit decay and loci expressed in ungerminated conidia. Total RNA was isolated from ungerminated conidia and decayed apple fruit infected with *P. expansum* R19 or *P. polonicum* RS1. There were 2442 differentially expressed genes (DEGs) between the R19 and RS1 in apple. Comparisons within species between apple and conidia revealed 4404 DEGs for R19 and 2935 for RS1, respectively. Gene ontology (GO) analysis revealed differential regulation in fungal transport and metabolism genes during decay, suggesting a flux in nutrient acquisition and detoxification strategies. In R19, the oxidoreductase GO category comprised 20% of all DEG groups in apple verses conidia. Ungerminated conidia from both species showed DEGs encoding the glyoxylate shunt and beta-oxidation, specifying the earliest metabolic requirements for germination. This is the first study to identify pre-loaded transcripts in conidia from blue mold fungi, reveal unique genes between species expressed during apple decay, and show the expression dynamics of known fungal virulence factors. These findings will enable development of targeted approaches for blue mold abatement strategies.

## 1. Introduction

*Penicillium expansum* is one of the most prevalent mycotoxigenic postharvest phytopathogens worldwide, causing severe damage to the pome fruit industry [1]. *P. expansum* and several other *Penicillium* species are the causal agents of blue mold decay, characterized by a soft, light brown, watery rot in the fruit [2]. *P. expansum* proliferates using wind- and water-borne asexual spores (conidia), which enter the host via wounds and natural openings (e.g., lenticels, stem ends, open sinus) to begin the infection process. The primary vegetative state of the fungus includes mycelial development, which, after extensive colonization, spreads across the apple surface in a tight radius and deep into the apple tissue. Although numerous *Penicillium* species cause blue mold, it is well established that *P. expansum* is the most prevalent and aggressive of these species. In contrast, *P. polonicum* causes much less decay and is quantitatively the weaker pathogen [3,4,5]. The observed discrepancies in decay between *P. expansum* and *P. polonicum* provides a unique opportunity to uncover the underlying transcriptional shifts that underpin virulence and the mechanisms that enable these fungi to successfully cause decay.

Virulence is a complex, multifactorial process driven by a variety of carefully orchestrated host–pathogen interactions. The molecules, toxins, genes, and signaling pathways associated with phytopathogenic fungal virulence mechanisms are abundant and include themes of redox regulation, CAZyme secretion, host recognition, and pH modulation [6,7]. The virulence molecules of interest in *P. expansum* include the mycotoxins patulin, citrinin, and penicillic acid [4,8]; degradative enzymes, such as polygalacturonases; other carbohydrate active enzymes (CAZymes) [9,10,11]; and secreted effectors [11,12]. Genes associated with these molecules and protein (e.g., biosynthesis, secretion) are essential for the establishment of successful infection by numerous phytopathogenic fungi during plant–pathogen interactions [13,14,15,16,17,18].

The sequencing, assembly, and annotation of fungal genomes coupled with modern molecular tools to facilitate discoveries in gene function allow the characterization of the signaling pathways and molecular regulators that modulate fungal virulence [16]. A variety of these genes have been identified in the postharvest phytopathogen *P. expansum* [19,20,21]. Among the genes that have been well characterized in *P. expansum* are those that encode transcription factors that modulate a variety of essential biological, developmental, and virulence functions, including LaeA, SntB, CreA, VeA, Ste12, PacC, and PeMetR [21,22,23,24,25,26,27,28]. Other relevant genes encode regulatory factors involved with reactive oxygen species (NoxR, NoxA, RacC), gluconic acid accumulation (GOX2), or vesicle-based secretion (Blistering1) [19,29,30], as well as genes encoding enzymes involved in the synthesis and regulation of secondary metabolites (PeRmtC, patulin gene cluster) [27,31,32]. Both reverse and forward genetic approaches have been implemented to determine the role of these genes in fungal virulence and apple fruit decay. Additionally, bioinformatic pipelines have been developed, in part, using transcriptomic data to identify and target select genes for determining their role in virulence [11,12].

The elucidation and characterization of the genes and pathways of *Penicillium* spp. that regulate and support virulence may yield novel and efficient approaches to control blue mold. Investigation into the differences in aggressiveness observed between *P. expansum* and *P. polonicum* can provide transcriptomic insights into the decay process that will lead to new management tools against *Penicillium* spp. that cause blue mold on pome fruits. The short-term goal of our study is to use a comparative transcriptome approach to identify loci, conserved pathways, and biochemical processes associated with ungerminated conidia and virulence in the blue mold fungi *P. expansum* and *P. polonicum*. Our long-term goal is to use these molecular data to guide the development of practical intervention strategies to reduce spore germination and or blue mold decay that can ultimately be commercialized to maintain fruit quality during routine long-term storage and transport.

## 2. Materials and Methods

### 2.1. Fungal Cultures, Isolate Propagation, and Apple Fruit Aquisition

*Penicillium expansum* (R19) was isolated in 2011 from a blue mold-infected ‘Red Delicious’ apple fruit in commercial cold storage in Carlisle, PA. Cultures of *P. polonicum* (RS1; originally identified as *P. solitum*) were obtained from a blue mold-infected apple in Oregon; the monosporial isolate was supplied by Dr. Bob Spotts [3,33,34]. Both single spore isolates were propagated on potato dextrose agar (PDA) in a temperature-controlled incubator at 25 °C with natural light levels.

Apples (‘Golden Delicious’, GD) were obtained from the Penn State Fruit Research and Education Center (Biglerville, PA, USA) and were assessed for maturity using a starch-iodine (I2KI) test as previously described [35]. All apples were at a later stage of maturity of an average 7–8 on the maturity scale (Cornell Starch Index Scale).

### 2.2. Characterization of Penicillium spp. Virulence in Apple Fruit

To assess the virulence of each fungal strain, ‘Golden Delicious’ apples (GD) were inoculated with conidial suspensions as previously described [10]. Briefly, apples were washed and then surface sanitized with 70% EtOH before puncturing the equator of the fruit with the tip of a sterile wounding tool (16 penny nail protruding from a 7.62 cm × 12.7 cm block of wood) to a depth and width of 3 mm to mimic stem punctures that occur during handling and storage. Conidial suspensions (10 µL of 1 × 10^5^/mL) of R19, RS1, or Tween-treated water (TTW) were pipetted into one wound/apple with ten replicates for each *Penicillium* species. Apples were stored in commercial cardboard boxes on compatible trays for 7 days at room temperature (21–23 °C) with lesion diameters measured 3, 5, and 7 days post-inoculation. This experiment was performed twice.

### 2.3. Sample Harvest and RNA Extraction from Penicillium spp. in Apple Fruit

The total RNA was extracted from *P. expansum* R19 and *P. polonicum* RS1 ramifying hyphae and actively growing mycelium from the leading edge of infected apple fruit. To obtain standardized inocula, PDA plates (25 mL) were inoculated with 100 µL of 1 × 10^4^ conidia/mL from RS1 or R19 isolates grown for 3 days at 25 °C. Four cultures for each isolate (RS1 or R19) were grown and spores from those plates were used to inoculate wounded GD apples as described above for the in vivo assay. Four GD apples were inoculated with each isolate and tissue containing fungi was harvested five days post-inoculation.

Total RNA was isolated from approximately 1500 mg of infected apple tissue. Specifically, the outer leading edge of the blue mold lesion was obtained (about 1 mm beyond visibly decayed tissue) and was frozen in liquid nitrogen (LN2) for immediate RNA extraction. Samples were ground with a mortar and pestle containing LN2 to a fine powder, and RNA was extracted according to the method described by Chang et al. [36]. Apple tissue samples were cleaned using the “Total RNA Extraction From Yeast” method outlined in the Qiagen RNeasy kit manual. RNA from fungi within decayed apple tissue was resuspended in DEPC-treated sterile water.

### 2.4. In Vitro Penicillium spp. Characterization

To characterize each strain in vitro, a conidial suspension (10 µL of 1 × 10^5^/mL) was pipetted onto Petri plates containing PDA and incubated at 25 °C in an incubator with natural light (Innova 450R). The germination percent of each strain was assessed 14 and 24 h post-inoculation using a light microscope to view spores deposited on the surface of PDA plates. Additional cultures of each isolate on PDA were maintained for 7 days with colony diameters measured and photographed 3, 5, and 7 days post-inoculation. These experiments were performed twice.

### 2.5. Sample Preparation and RNA Extraction from Ungerminated Conidia

Total RNA was also extracted from *P. expansum* R19 and *P. polonicum* RS1 ungerminated conidia obtained from sporulating cultures in vitro. First, PDA plates (25 mL) were inoculated with 100 µL of 1 × 10^4^ conidia/mL from RS1 or R19 isolates grown for 3 days at 25 °C. Four cultures for each isolate (RS1 or R19) were grown and spores from those plates were used to inoculate PDA. Conidia (R19 and RS1) were harvested eleven days after initial plating by adding 1 mL of TTW to the PDA plate and gently scraped with a sterile plastic spreader. Conidial suspensions were aspirated from the surface of the plate using a sterile pipette tip and deposited into 1.5 mL Eppendorf tubes. Spores were then placed in a microcentrifuge for 5 min at 14,000× *g* and pelleted. After removing supernatant, 1 mL of TTW was added and the spores were vortexed (Vortex-Genie2, Scientific Industries, New York, NY, USA) for 10 s on the highest setting to mix. This process was repeated two times and the resulting suspension was visualized using a compound microscope with 10 µL of suspension placed on a glass slide. Several fields of view were observed and contained copious amounts of spores with no mycelial fragments, swollen spores, or germ tubes visualized.

To extract RNA, conidia were immediately homogenized with a Bead Mill Ruptor 24 (Omni) at 3000 RPM for 3 one-minute cycles. In between cycles, samples were removed from the machine and chilled on ice for 5 min. The Trizol RNA extraction method (Invitrogen), with an additional gDNA contamination removal step using TURBO DNase (Invitrogen), was used according to the manufacturer’s protocol for conidia [37]. Each RNA sample was resuspended in DEPC-treated sterile water.

### 2.6. RNA-Sequencing

The sample quality of RNA samples was assessed using gel electrophoresis (1% agarose gel, 75 v for 35 min), a Nanodrop spectrophotometer (Thermofisher Scientific, Wilmington, DE, USA) with 260 nm/280 nm and 260 nm/230 nm absorbance ratios, and an Agilent 2100 Bioanalyzer for RNA integrity number (RIN ≥ 8.0) at the Beijing Genomics Institute (BGI, Hong Kong, China). RNA sequencing, also conducted at BGI, was performed using the short read DNBSeq platform (BGISEQ-500) for paired end 2 × 150 bp reads.

### 2.7. Bioinformatic Analyses and Differential Gene Expression

The quality and quantity of the RNA-sequenced reads were examined using FASTQC analysis [38]. The transcriptomes were assembled de novo via Trinity on Galaxy (usegalaxy.org), using default settings and a flag for read trimming. To confirm assembly completeness, rnaQUAST was implemented before the final quantification of transcripts with Salmon [39]. The prefiltering of low count genes (≤10) was performed with apeglm (Bioconductor, Boston, MA, USA) for LFC shrinkage [40]. Differential gene expression (DEG) analysis (log_2_ fold change ≥ 2, *p*-value < 0.05) was completed in R using DESeq2 [40,41] with the GenomicFeatures, tximport and tximportData packages. The visualization of the data was performed in R with EnhancedVolcano, ggplot2, and Venny 2.1 [42]. A list of genes known or hypothesized to play a role in virulence was generated (Table S1) and DEGs from this list were visualized as heat maps using ggplot2.

### 2.8. Gene Ontology and KEGG Mapping

The annotation database, Gene Ontology Terms, was used to add GO terms to the *Penicillium expansum* MD-8 genome (GenBank Accession: GCA_000769745.1) via Blast-2-GO (Biobam Bioinformatics Solutions, Valencia, Spain). The gene ontology (GO) was performed using the topGO program in R (Bioconductor, package version 2.46). Genes differentially expressed (log_2_ fold change ≥ 2, *p*-value < 0.05) between each comparison were included in the analysis. Fisher’s exact test was used with weight01 algorithm for statistical analyses for each comparison. Results were focused using the biological processes (BP) category, and all results with a *p*-value < 0.01 were considered significantly enriched for this analysis and were visualized via Excel (Microsoft). Additionally, genes from the RNA-Seq data with log_2_ fold change ≥ 2 from each comparison above were also given KO numbers using the BlastKOALA function in KEGG. Images were generated from the KEGG Mapper tool for select biological pathways [43].

### 2.9. Statistical Analysis

Statistical analyses for the virulence and in vitro characterization assays were performed using a Levene’s test to determine homogeneity between samples. The data were not normally distributed, so a Kruskal–Wallis test with post hoc Pairwise Wilcoxon test using Benjamini–Hochberg *p*-value adjustment was used.

## 3. Results

### 3.1. Disparities in Decay Exist between Two Penicillium spp. in Apple Fruit

**Figure 1:**
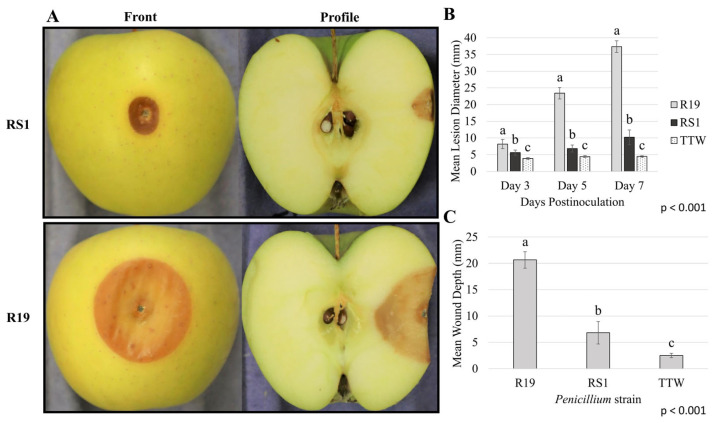
Differences in decay caused by *Penicillium expansum* (R19) and *Penicillium polonicum* (RS1) in ‘Golden Delicious’ apple fruit. (**A**) Representative blue mold lesion front (left) and profile (right) of inoculated *P. polonicum* RS1 (top) or *P. expansum* R19 (bottom). (**B**) Mean lesion diameters of R19 and RS1 after 3, 5, and 7 days post-inoculation in decayed apple fruit. (**C**) Mean wound depth of R19 and RS1 7 days post-inoculation. For TTW, no decay was observed, and the diameter of the wound was recorded. Error bars represent the standard deviation, with samples from two independent trials. Different letters on the bar graphs denote statistical significance (*p* < 0.001), which was determined using a Kruskal–Wallis test and a post hoc pairwise Wilcoxon test with a Benjamini–Hochberg *p*-value adjustment. TTW = Tween Treated Water (negative control).

**Figure 2:**
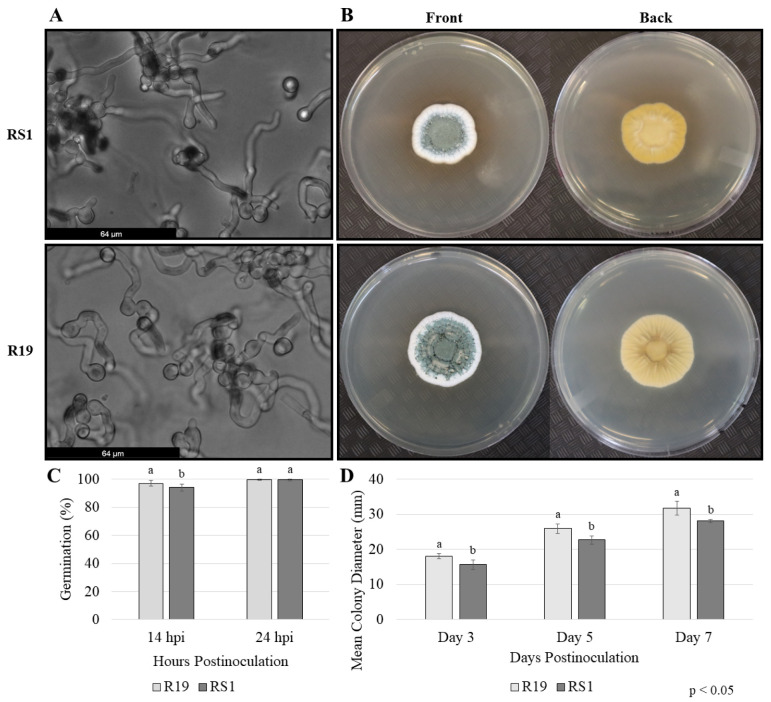
Conidial germination, mycelial growth, and morphology *P. expansum* R19 and *P. polonicum* RS1 on Potato Dextrose Agar. (**A**) Germinated conidia at 14 h and 24 h after inoculation. (**B**) Radial growth 7 days after inoculation on Petri plates containing PDA. (**C**) Conidial germination 14 h and 24 h post-inoculation for each strain. (**D**) Mean colony diameters were measured 3, 5, and 7 days post-inoculation on Petri plates containing PDA. Conidial germination was determined by considering only spores with a germ tube at least 2.5× the size of the conidia. Bars represent the mean of three biological replicates from two trials, and error bars were generated using standard deviation. Different letters on bars denote statistical significance (*p* < 0.05), which was determined using a Kruskal–Wallis test and post hoc pairwise Wilcoxon test with Benjamini–Hochberg *p*-value adjustment. Hpi = hours post-inoculation.

To quantify previously observed differences in virulence between *P. expansum* R19 and *P. polonicum* RS1, apple fruits were inoculated with an identical volume and concentration of spores from each isolate (Figure 1). On average, R19 caused significantly more decay with lesions measuring 37.4 mm in diameter and 20.7 mm deep 7 days post-inoculation. During the same incubation period, RS1 produced lesions with a mean diameter of 10.2 mm and 6.8 mm deep, i.e., lesions smaller by 72.8% than those produced by R19. To compare fungal germination and mycelial growth between the two strains, conidial germination was monitored after 14 and 24 h; radial growth was measured 3, 5, and 7 days post-inoculation (Figure 2). Spore germination percentages were nearly identical between R19 and RS1, with 97.2% and 94.2% average germination, respectively, after 14 h, and full germination and initial colony formation by 24 h, respectively. After 7 days, RS1 showed a slightly smaller colony with an irregular leading edge when compared to the larger, more circular, and uniform appearance of the colony formed by the R19 isolate. The mean colony diameter of R19 colonies was an average of 32.2 mm, while RS1 had an average of 28.1 mm, representing a 12.8% difference in colony diameter between the two isolates.

### 3.2. Transcriptomic Differences Were Elucidated between Ungerminated Conidia and Fungal Mycelium during Apple Fruit Decay

RNA-Seq was performed on samples extracted from the leading edge of an apple fruit that was wound-inoculated with *P. expansum* R19 or *P. polonicum* RS1 and also from ungerminated conidia of each strain in vitro. Genome-guided Trinity de novo transcriptome assembly, where transcripts are utilized as sequenced, was used to capture true variation between samples [44]. The transcriptome analysis resulted in an average of 83,507 transcripts per sample, the mean number of transcripts >1000 bp was 26,885, the average number of GeneMarkS-T genes was 45,107, and the average database coverage of 67.9% reported from the Trinity summary. The mean number of unaligned transcripts was 67,635 and the average number of mismatches per transcript was 1.718. Significantly differentially expressed genes were analyzed as R19 over RS1, which allows the observation of the expression patterns of each individual isolate during the different life stages, e.g., during decay or as ungerminated conidia. The gene expression of select loci was plotted across life stage and strain.

**Figure 3:**
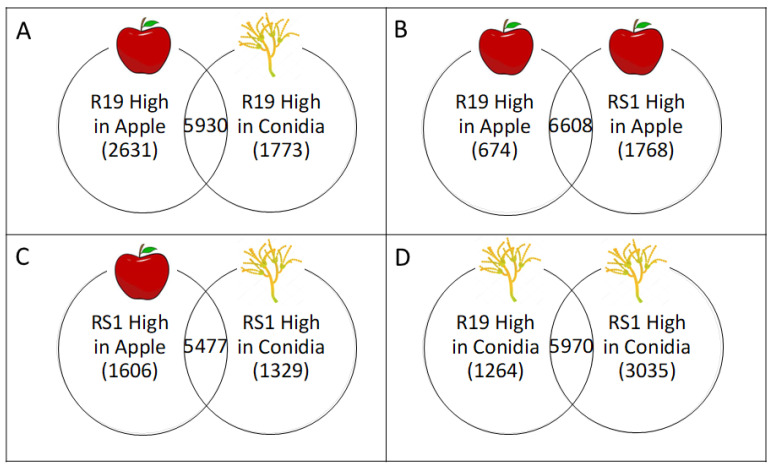
Venn diagram of differentially expressed genes (DEGs) between *Penicillium* spp. R19 and RS1 in apple and conidia. Comparisons show DEGs higher in each category between (**A**) R19 apple vs. ungerminated conidia, (**B**) Ramifying hyphae and mycelium in apple from R19 and RS1, (**C**) RS1 ramifying hyphae and mycelium in apple vs. ungerminated conidia, and (**D**) ungerminated conidia from R19 and RS1. Numbers represent upregulation (high) expression (log_2_ fold change ≥ 2) with *p*-value < 0.05. Values in overlapping regions are genes that did not have significant differential gene expression between sample types.

**Figure 4:**
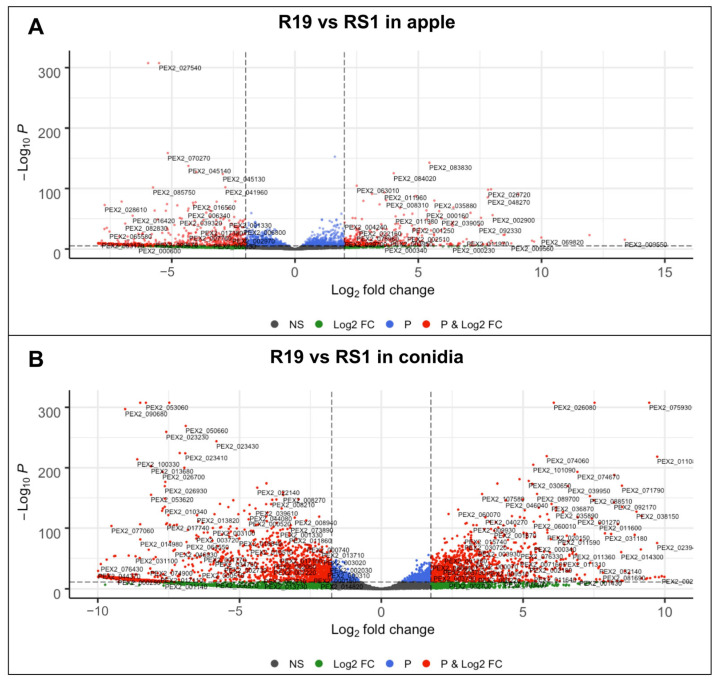
Volcano plots of differential gene expression between *Penicillium* spp. R19 and RS1. Differential gene expression in R19 and RS1 from (**A**) ramifying hyphae and mycelium in apple fruit and (**B**) ungerminated conidia. Green = log_2_ fold change, black = not significant, *p* = significant *p*-value (*p* < 0.05), and red = log_2_ fold change with a significant *p*-value (*p* < 0.05). Dotted lines indicate log_2_ fold change ≥2 threshold.

After QC and the alignment of the reads to the R19 genome, log2 fold changes in gene expression from each dataset (R19 and RS1 from apple or conidia) were determined. Total gene counts for differentially expressed genes (DEGs; log2 fold change ≥ 2, *p* < 0.05) show an overall higher number of DEGs for R19 and RS1 during apple fruit decay as compared to ungerminated conidia (Figure 3). Overall, R19 has fewer unique DEGs than RS1 in both decayed apple and conidia, with, respectively, 674 and 1768 DEGs in apple and 1264 and 3035 DEGs in conidia (Figure 3 and Figure 4). Between sample types, both R19 and RS1 had higher numbers of DEGs during apple fruit decay than in ungerminated conidia, with 2631 and 1606 DEGs in apple, respectively, and 1773 and 1329 DEGs in conidia.

### 3.3. Overlapping and Distinct Biochemical Themes Are Evident in Different Life Stages Examined in Two Penicillium spp.

**Figure 5:**
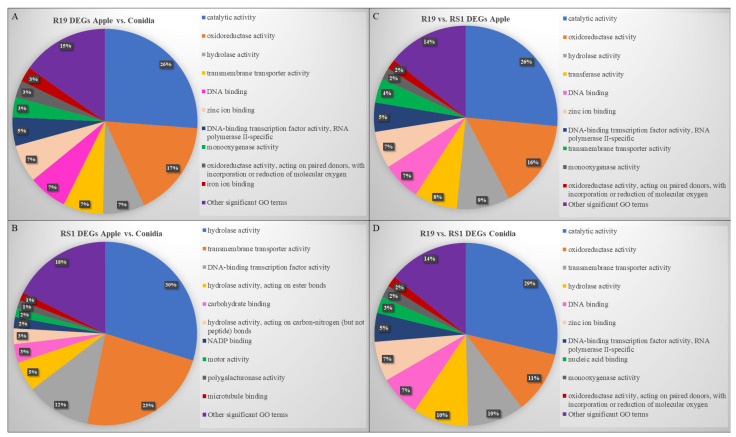
Top 10 molecular functions of differentially expressed genes (DEGs) in *Penicillium* spp. R19 and RS1 from ramifying hyphae and mycelium in apple and ungerminated conidia. Gene ontology analysis of (**A**) R19-decayed apple and ungerminated conidia DEGs, (**B**) RS1-decayed apple and ungerminated conidia, (**C**) R19- and RS1-decayed apple, and (**D**) R19 and RS1 ungerminated conidial transcripts. Gene ontology categories within the molecular functions subcategory are shown with the highest 10 categories for DEGs listed from each comparison. Gene ontology statistics performed using Fisher’s exact test and the weight01 algorithm (*p*-value < 0.01).

Gene ontology was performed on each RNA-Seq comparison (R19 in apple vs. conidia, RS1 in apple vs. conidia, R19 vs. RS1 from apple, R19 vs. RS1 from conidia) to determine the highly regulated categories within the DEGs (Figure 5, Table S2). Molecular functions with a *p*-value < 0.05 were deemed significantly regulated between each comparison. When evaluating the R19 apple and conidia samples, 56 molecular function categories were significantly regulated with a summation of 4079 genes across all categories. Similarly, the RS1 apple and conidia comparison showed 46 groups significantly regulated with 760 genes falling into discrete categories. For comparisons between R19 and RS1 in apple, there were 2391 genes in 41 groups and 4452 genes in 46 categories were differentially regulated from ungerminated conidia.

Common biochemical themes arose within the top ten categories for each comparison (Figure 5). These themes include hydrolase activity (7% for R19 in apple vs. conidia, 35% for RS1 in apple vs. conidia, 9% in R19 vs. RS1 in apple, and 10% in R19 vs. RS1 conidia), and transmembrane transport (7% for R19 in apple vs. conidia, 23 for RS1 in apple vs. conidia, 4 in R19 vs. RS1 in apple, and 10% in R19 vs. RS1 conidial samples). Catalytic activity was the top category in all but the RS1 apple vs. conidia DEG comparison; hydrolase activity was the top category for the RS1 apple vs. conidia. In every case, the ‘other significant genes’ category was 14–15% of total significant categories, indicating a large majority of the DEGs fell into groups belonging to the top 10 previously discussed areas.

**Figure 6:**
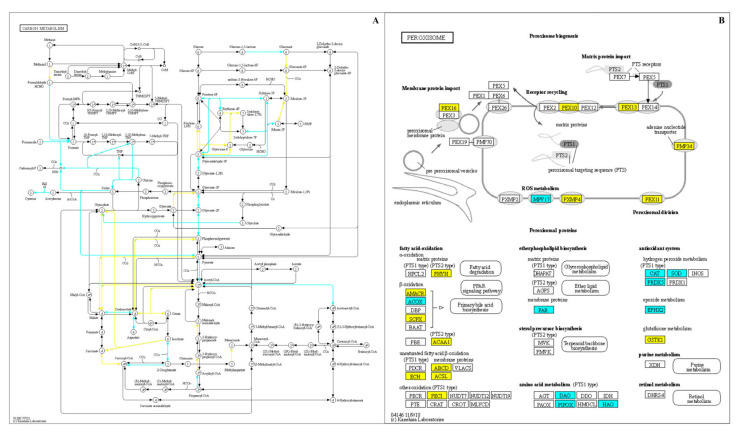
Kyoto Encyclopedia of Genes and Genomes (KEGG) maps of select metabolic pathways showing differentially expressed genes in the highly virulent strain *Penicillium expansum* R19. Select genes differentially regulated (log_2_ fold change ≥2, *p* < 0.05) in R19 between ramifying hyphae and mycelium from apple and ungerminated conidia. DEGs comprising (**A**) carbon metabolism and (**B**) peroxisomal biochemical functions are highlighted in yellow (high in ungerminated conidia) or cyan (high in decayed apple). Images generated using Kegg.com.

The Kyoto Encyclopedia of Genes and Genomes (KEGG) was implemented to determine patterns in the DEG categories and to map differentially regulated transcripts into distinct biochemical pathways, specifically for the highly aggressive R19 strain from each sample type. The DEGs from the R19 conidia vs. apple comparison were assigned KO numbers using BlastKOALA. Then the KEGGmapper program was used to allot the DEGs to specific biochemical pathways, which included general carbon metabolic and peroxisome pathways involving redox regulation (Figure 6). In the carbon metabolism pathway, preloaded transcripts from ungerminated conidia were enriched for portions of carbon metabolism, such as the glyoxylate shunt, which were less expressed in the later stages of R19-mediated infection. Similarly, the peroxisome pathways showed an upregulation of genes in R19 conidia involved in fatty acid oxidation that was reduced during apple fruit decay. When examining both carbon metabolism and peroxisomal pathways in decayed apple samples (Figure 6), increased expression in amino acid metabolism genes was observed in both KEGG maps. R19 also showed an upregulation of genes from the decayed apple tissue samples involved in hydrogen peroxide metabolism.

### 3.4. Fungal Virulence-Associated Genes Encode a Variety of Biochemical Categories

**Figure 7:**
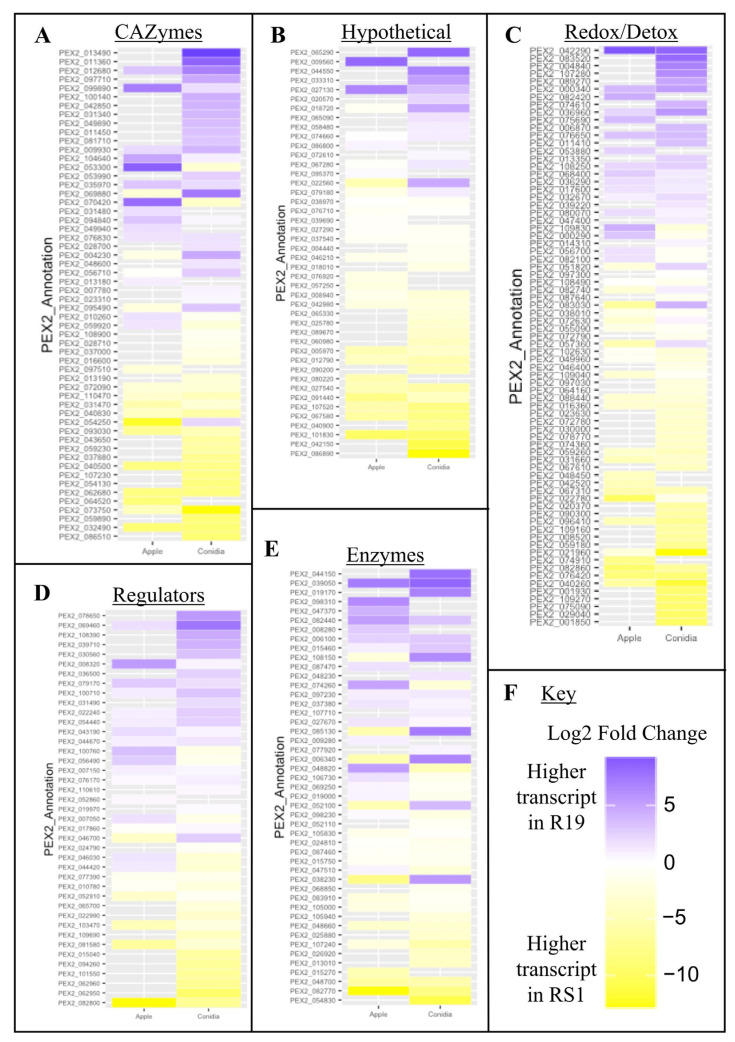
Heat maps showing differentially expressed genes encoding known or predicted virulence factors across ungerminated spores and ramifying hyphae and mycelium from decayed apple fruit from highly virulent (R19—*Penicillium expansum*) and weakly aggressive strains (RS1—*P. polonicum*). Genes previously identified as virulence factors depicted with annotations (PEX2_Annotation) and sorted into (**A**) CAZymes; (**B**) hypothetical proteins and genes with unknown function; (**C**) redox and detoxification-related genes; (**D**) regulators, including transcription factors; and (**E**) enzymes. (**F**) Key to heat map: colors represent log_2_ fold change between the R19 and RS1 samples, with blue showing higher expression in R19 samples and the yellow showing higher expression in RS1. The left-side columns show the log_2_ fold change between ramifying hyphae and mycelium from apple while the right-side columns show the log_2_ fold change between ungerminated conidia.

A list of 302 known or hypothesized *P. expansum* virulence factor-encoding genes was curated from the literature, including loci that result in reduced apple fruit decay when mutated or deleted in *P. expansum* (Table S1). Heat maps were generated to compare gene expression profiles between R19 and RS1 to find genes associated with fungal aggressiveness, as well as to determine expression profiles in ungerminated conidia (Figure 7). Each gene was grouped into a category based on predicted biochemical moiety/domain or by a known function identified by deletion analysis causing reduced apple fruit decay. Across all functional categories, the highest number of DEGs was between samples from the ungerminated conidia of the weakly virulent (RS1) and highly virulent (R19) species. In the CAZymes category from apple tissue, there were nine genes that were high in R19 with either no expression shift or a low expression for RS1. For the hypothetical category, there were only two genes upregulated in R19 apple compared to those high in RS1, PEX2_009560 and PEX2_027130. However, numerous genes in R19 were high while RS1 showed no change, including hypotheticals and peptidoglycan-binding lysins (LysM proteins). In the redox/detox group, the comparisons showed fifteen genes that were high in R19 apple with no DEG in RS1, including six CYP51 and oxidase-annotated genes. The regulator class included a transcription factor that was high in R19 apple and high in RS1 conidia, as well as eight genes, mostly phosphoesterase and carboxylesterase-encoding genes, that were also elevated in R19 apple. R19 had three genes encoding glucose methanol choline oxidoreductases that were high in conidia. R19 also showed greater expression for lipases and a chorismite mutase during apple tissue decay compared to RS1.

## 4. Discussion

The blue mold of apple fruit is a globally relevant problem for the pome fruit industry. *Penicillium* decay manifests not only as food loss but can also result in patulin contamination which threatens human health. Our comparative approach used the highly virulent *Penicillium expansum* R19 and the weakly aggressive *Penicillium polonicum* RS1 to uncover new, and confirm known, mechanisms mediating fungal virulence. Both species possessed quantifiable differences in their ability to cause blue mold decay in apple fruit [5]. Our study confirmed that *P. expansum* R19 is quantitatively more virulent than *P. polonicum* RS1 with 72.8% larger lesions in apple fruit over the same incubation time. The general capacity of the two strains to achieve optimum fungal growth in vitro was similar, with colony diameters only 12.8% larger in R19 and conidial germination rates nearly identical. Thus, observed differences in virulence between the two strains reflect aspects of fungal–host interactions and are not solely the result of differing fungal growth capacities, highlighting a unique opportunity to explore gene networks and signaling pathways involved in *Penicillium* post-harvest virulence.

Transcriptomics provides an ideal tool for better understanding the biological systems and networks that control fungal survival and virulence. Previous research has investigated *P. expansum* transcription in different contexts, including the process of pear fruit decay [45]; spore germination in vitro [46]; the early stages of apple fruit infection [47]; and comparisons between decay and environmental pH [48]. By leveraging a comparative RNA-Seq tactic for R19 and RS1, we revealed insights into the biochemical mechanisms, pathways, and genes in both blue mold fungi during apple infection and pre-germination events in conidia. Among the many differences between the two *Penicillium* species were numerous transcriptional regulators, oxidoreductases, hydrolases, and transmembrane transporters. Across each comparison, transport and transporter components were highly and differentially regulated. Transporters are routinely involved in nutrient acquisition. Thus, the vast differences between the metabolic states of the different samples (ungerminated conidia vs. ramifying hyphae and mycelium in apple fruit) reflect transcriptional shifts observed in transporters and other DEGs identified from general carbon metabolism and other metabolic pathways associated with nutrient acquisition by the fungus.

Transporters mediate many functions outside of nutrient uptake, such as host–parasite interactions, increasing tolerance to xenobiotics, and the modulation of antimicrobial resistance (AMR). For example, previous transcriptomic and functional genetic research has shown that fungicide resistance in strains of major post-harvest apple pathogens (e.g., *Penicillium* spp., *Botrytis cinerea*) is mediated by differential regulation and coordinated expression of MDR, MFS, and ABC transporters [49,50,51,52]. Furthermore, mycotoxin tolerance (e.g., patulin) is mediated by efflux pumps. Their inhibition resulted in increased sensitivity of *Penicillium* spp. to exogenously applied purified patulin [35]. *P. expansum* R19 contains an intact 40kb patulin gene cluster and produces patulin in vitro and in vivo, while RS1 lacks a complete cluster and thus does not produce patulin. This difference in toxigenic potential may indicate why we observed the increased differential expression of transporters in the R19 dataset compared to RS1 during apple fruit decay, as the R19 strain may rely on these transporters to export toxin produced in order to avoid self-toxicity [5,53]. Moreover, transport is also impacted by the apple phenolic, quercetin, which demonstrates one part of the apple fruits influence on fungal transcriptional regulation [54]. Further studies using functional analysis, drug inhibition/activation studies, and/or metabolite tagging are needed to determine which specific transporters or classes (ATP-binding cassette, major facilitator family) are involved in these interactions.

Interestingly, when comparing R19 and RS1 in apple, glutathione-associated genes were shown to be regulated within the gene ontology analysis. Glutathione is a non-enzymatic antioxidant important in fungi, plants, and animal systems [55]. In addition to providing oxidative homeostatic balance in cells via radical scavenging, glutathione plays a central role in patulin detoxification within human cell lines and in rat models [56,57,58]. With lower glutathione pools from binding patulin, however, the cell cannot adapt and quench oxygen radicals and thusly encounters high oxidative stress that manifests in cell death. Additionally, patulin increases lipid peroxidation [56,57,58]. The mode of action for patulin detoxification in fungi and plants has yet to be elucidated, but the presence of patulin in R19, yet not RS1, could be one reason for these transcriptional shifts in redox activity. Enzymatic antioxidants are also critical in fungal systems for maintaining redox in response to host defenses. Detoxification-associated genes involved in hydrogen peroxide biosynthesis and sequestering and, for example, were expressed in fungal-infected apple samples, suggesting their importance during the decay process. Hydrogen peroxide and other reactive oxygen species (ROS) are highly conserved plant defense responses against pathogens, so the tightly regulated, highly coordinated expression of these fungal genes while in apple are likely critical for blue mold survival and proliferation [59].

Many groups have identified and characterized *Penicillium expansum* virulence factors in order to understand the molecular mechanisms behind blue mold decay. From the published literature, we generated a comprehensive list of the genes associated with virulence in apple fruit, encompassing both those predicted to impact decay based on bioinformatic programs and those experimentally verified to have a reduced phenotype in apple fruit [11,12]. Unfortunately, not all of the bioinformatically predicted virulence factors were observed to be differentially regulated, which may reflect on the predictive process of those in situ methods. However, it is also likely that the identified virulence factors are regulated at different levels/times throughout the decay process in a highly coordinated, orchestrated manner. Further, the identified virulence factors may not be exclusively involved in the decay process. Some belong to other pathways for metabolism or stress responses that also cross network with the decay process that manifests in the level of fungal aggressiveness. It is known that some transcriptional regulators that impact fungal virulence are global regulators important for primary and secondary metabolism beyond mycotoxin production, including PeMetR, LaeA, VeA, SntB, PacC, and CreA [7,21,22,25,26,60]. Carbon catabolism, regulated by CreA, has been linked to pH modulation via organic acid production and is also critical for full virulence in *P. expansum* [7,23]. Blistering1, shown to impact extracellular patulin accumulation, pH modulation, and proper protein secretion, also impacts vesical-based transport and cell wall-degrading enzyme activities [19]. Hence, the genes, pathways, and processes associated with virulence and apple fruit colonization are very complex, play multiple roles in the biology of the fungus, and may not be completely uncoupled from the process of decay. Therefore, the temporal activation of those genes may be outside the scope of the two timepoints presented in this study, as the gene expression of *P. expansum* is altered even within the first few hours while in the apple host [47].

While some of the virulence factors did not show differential regulation within the scope of this study, a large portion did show altered gene expression between fungal species. For example, nine genes annotated from the CAZyme category were shown to be higher in R19 than RS1 in apple. As has been shown in previous reports and confirmed in this study, *P. expansum* creates larger lesions in apple than the *P. polonicum* counterpart [5]. These CAZymes upregulated or present in *P. expansum* exclusively could be a major reason for this difference. Additionally, some CAZymes are host-specific in activity (i.e., apple, pear, or others) [61], which may be why *P. polonicum*, while having a broader host range, did not have those same genes upregulated in this study. Lesion formation in the host has also been shown to be due to patulin production [35]. Interestingly, R19 has complete gene clusters for both, whereas RS1 only has partial biosynthetic gene clusters for each [53]. This could also contribute to lesion formation being larger for *P. expansum*.

In addition to focusing on virulence during decay, our study is the first to profile pre-loaded transcripts in ungerminated spores in two *Penicillium* spp. Amongst the genes identified and quantified from the ungerminated conidia were those involved in the fatty acid metabolism that occurs in peroxisomes, specifically β-oxidation and the glyoxylate shunt of the TCA cycle [62]. Here we show that these components are present in the ungerminated spores prior to germination. We hypothesize that spores need to utilize stores of lipids as a temporary energy source to fuel cell wall biogenesis, germ tube elongation, and growth until carbon sources are liberated from the host after hyphae have begun to secrete CAZymes and uptake nutrients. Interestingly, polyketide mycotoxin production has also been associated with the regulation of β-oxidation in the fungal cell [63], although further investigation is warranted on whether the DEGs observed in this study are involved with patulin production in these *Penicillium* strains. Nevertheless, our observations are consistent with what is already known about early fungal germination and development stages in other fungi [64].

Previous works have compared *P. expansum* R19 to *P. polonicum* RS1 (previously *P. solitum*) through whole genome sequencing and phylogeny, and it has been shown that the two species are closely related compared to others in the genus [5,33]. Moreover, using the antiSMASH program, Wu et al. [5] showed there are 66 predicted secondary metabolite clusters in *P. expansum*, compared to 58 predicted clusters in *P. polonicum*. Additional comparisons have been made regarding volatile organic compounds (VOCs) to specifically study differences in oxylipins [53]. Surprisingly, the less virulent RS1 produced more oxylipins than the more virulent R19, demonstrating that virulence in apple fruits and toxicity in other contexts do not always coincide. Our experimental data in apples showed 16 DEGs between R19 and RS1 in the ‘dioxygenase’ GO category (molecular function), supporting the differences predicted by Yin et al. [53] from bioinformatics analysis. Our transcriptomic work complements these datasets regarding their mycotoxigenic profiles and VOC-producing capability, providing a holistic perspective about the comparative virulence of these two blue mold pathogens. However, the specific role of oxylipins in fungal virulence during apple fruit decay has not been clarified and should be investigated in the future.

## 5. Conclusions

The postharvest storage environment is constantly challenged by the presence of *Penicillium* spp. that can cause decay and mycotoxin accumulation in food stuffs. The goal of this study was to determine the genes expressed by two blue mold fungi, both from ungerminated conidia and ramifying hyphae and mycelium in decayed apple fruit. We identified and quantified transcripts from ungerminated spores before spore germination occurred. We also determined transcriptional differences between the two blue mold fungi during advanced stages of decay, such as enzymatic/CAZyme expression and global regulation shifts, including some known virulence regulators. Further research efforts to integrate omics-based approaches from a systems viewpoint (proteomic, metabolomic, VOComic) will be key to determining the full requirements of blue mold fungi during apple fruit colonization and decay. Ultimately, this knowledge can be used to mitigate blue mold via the disruption of processes important for decay and/or by interfering with conidial germination. These molecular insights can be exploited to maintain fruit quality and improve food safety by minimizing patulin contamination. Our work not only provides seminal findings concerning *Penicillium* virulence but also identifies the putative genes and pathways in ungerminated conidia that can be the subject of a new class of sporulation inhibitors and decay control strategies.

## Data Availability

The dataset supporting the conclusions of this article is available in the NCBI Gene Expression Omnibus (GEO) repository with accession GSE209987, or at the following link: https://www.ncbi.nlm.nih.gov/geo/query/acc.cgi?acc=GSE209987. *Penicillium expansum* genome available in NCBI GenBank used for transcriptome mapping: GCA_000769745.1.

## Supplementary Materials

The following supporting information can be downloaded at: https://www.mdpi.com/article/10.3390/microorganisms10122414/s1, Table S1: Virulence factors of *Penicillium* spp.; Table S2: List of significant GO terms associated with the number of DEGs for each sample comparison.
