# Supplementary material for: Comparative Penicillium spp. Transcriptomics: Conserved Pathways and Processes Revealed in Ungerminated Conidia and during Postharvest Apple Fruit Decay
Source: Microorganisms. 2022 Dec 6;10(12):2414. doi: 10.3390/microorganisms10122414 (PMC9788453; doi:10.3390/microorganisms10122414)
Supplement: Supplementary file 1 [file microorganisms-10-02414-s001.zip › microorganisms-2065614-supplementary.pdf]

## Supplementary Figures and Tables

**Table S1.** Virulence factors of *Penicillium* spp.

| # | PEX2_ID Annotation | Annotation | Encoded Protein Name | Reference |
|---|---|---|---|---|
| 1 | PEX2_058480 | Hypothetical protein | PeMetR | 25 |
| 2 | PEX2_022240 | Zinc finger, C2H2 | LaeA | 22 |
| 3 | PEX2_043190 | Velvet factor | VeA | 26, 27 |
| 4 | PEX2_087460 | Protein arginine N-methyltransferase PRMT5 | PeRmtC | 32 |
| 5 | PEX2_025780 | Peptidoglycan-binding Lysin subgroup | LysM12 | 11 |
| 6 | PEX2_101830 | Peptidoglycan-binding Lysin subgroup | LysM15 | 11 |
| 7 | PEX2_027670 | Peptidase S8/S53, subtilisin/kexin/sedolisin | S8-PePRT | 12 |
| 8 | PEX2_044670 | Zinc finger, C2H2 | Ste12 | 28 |
| 9 | PEX2_053880 | Riboflavin synthase-like beta-barrel | NoxA | 30 |
| 10 | PEX2_056490 | Tetratricopeptide-like helical | NoxR | 30 |
| 11 | PEX2_019970 | Small GTPase superfamily, Rab type | RacA | 20 |
| 12 | PEX2_022240 | Zinc finger, C2H2 | CreA | 23 |
| 13 | PEX2_110610 | Zinc finger, PHD-type | SntB | 21 |
| 14 | PEX2_008940 | Heat shock protein, DNAJ domain | Blistering1 | 19 |
| 15 | PEX2_077390 | PacC transcription factor | PacC | 24 |
| 16 | PEX2_082770 | Glucose-methanol-choline oxidoreductase, C-terminal | PatE | 31 |
| 17 | PEX2_031340 | Glycoside hydrolase, family 28 | PEPG1 | 9 |
| 18 | PEX2_080220 | Necrosis inducing protein | NLP1 | 12 |
| 19 | PEX2_064520 | Glycoside hydrolase, superfamily | | 11 |
| 20 | PEX2_052250 | hypothetical protein PEX2_052250 | | 11 |
| 21 | PEX2_052260 | hypothetical protein PEX2_052260 | | 11 |
| 22 | PEX2_031470 | Pectin lyase fold/virulence factor | | 11 |
| 23 | PEX2_031480 | Pectin lyase fold/virulence factor | | 11 |
| 24 | PEX2_031490 | Exonuclease, RNase T/DNA polymerase III | | 11 |
| 25 | PEX2_050990 | Glycoside hydrolase, family 35 | | 11 |
| 26 | PEX2_051820 | FAD linked oxidase, N-terminal | | 11 |
| 27 | PEX2_052100 | Catalase, mono-functional, heme-containing | | 11 |
| 28 | PEX2_052110 | Six-hairpin glycosidase | | 11 |
| 29 | PEX2_031660 | Short-chain dehydrogenase/reductase SDR | | 11 |
| 30 | PEX2_103470 | Carboxylesterase, type B | | 11 |
| 31 | PEX2_103620 | Beta-hexosaminidase subunit alpha/beta | | 11 |
| 32 | PEX2_104640 | Glycoside hydrolase, superfamily | | 11 |
| 33 | PEX2_105000 | Carbohydrate-binding WSC, subgroup | | 11 |
| 34 | PEX2_105830 | Proprotein convertase, P | | 11 |
| 35 | PEX2_105940 | Concanavalin A-like lectin/glucanase, subgroup | | 11 |
| 36 | PEX2_055830 | Cytochrome P450, E-class, CYP52 | | 11 |

| | | | | |
|---|---|---|---|---|
| 37 | PEX2_027670 | Peptidase S8/S53, subtilisin/kexin/sedolisin | | 11 |
| 38 | PEX2_026690 | Carboxylesterase, type B | | 11 |
| 39 | PEX2_026920 | Lipase, class 3 | | 11 |
| 40 | PEX2_027130 | Lipopolysaccharide-modifying protein | | 11 |
| 41 | PEX2_027290 | Lipopolysaccharide-modifying protein | | 11 |
| 42 | PEX2_027540 | Ves allergen | | 11 |
| 43 | PEX2_037850 | hypothetical protein PEX2_037850 | | 11 |
| 44 | PEX2_036960 | FAD linked oxidase, N-terminal | | 11 |
| 45 | PEX2_037000 | Glycoside hydrolase, family 7 | | 11 |
| 46 | PEX2_037380 | Peptidyl-prolyl cis-trans isomerase, FKBP-type | | 11 |
| 47 | PEX2_037540 | Heat shock protein DnaJ, N-terminal | | 11 |
| 48 | PEX2_095370 | hypothetical protein PEX2_095370 | | 11 |
| 49 | PEX2_095490 | Glycoside hydrolase, family 28 | | 11 |
| 50 | PEX2_094260 | Histidine phosphatase superfamily, clade-2 | | 11 |
| 51 | PEX2_094770 | EGF-like calcium-binding | | 11 |
| 52 | PEX2_094840 | Glycoside hydrolase, family 28 | | 11 |
| 53 | PEX2_096410 | Cytochrome P450 | | 11 |
| 54 | PEX2_106730 | DNA glycosylase | | 11 |
| 55 | PEX2_032490 | Acyl transferase/acyl hydrolase/lysophospholipase | | 11 |
| 56 | PEX2_008520 | Cytochrome P450 | | 11 |
| 57 | PEX2_009280 | Peptidase aspartic, catalytic | | 11 |
| 58 | PEX2_009560 | Protein of unknown function DUF1365 | | 11 |
| 59 | PEX2_009930 | Alpha/beta hydrolase fold-3 | | 11 |
| 60 | PEX2_010260 | Glycoside hydrolase, superfamily | | 11 |
| 61 | PEX2_007290 | Pectate lyase, catalytic | | 11 |
| 62 | PEX2_006870 | Multicopper oxidase, type 2 | | 11 |
| 63 | PEX2_007050 | Glutamyl/glutaminyl-tRNA synthetase, class Ib | | 11 |
| 64 | PEX2_007150 | Transcription factor, fungi | | 11 |
| 65 | PEX2_007780 | Alpha/beta hydrolase fold-3 | | 11 |
| 66 | PEX2_008280 | Galactosyl transferase | | 11 |
| 67 | PEX2_008300 | hypothetical protein PEX2_008300 | | 11 |
| 68 | PEX2_008320 | Transcription factor, fungi | | 11 |
| 69 | PEX2_065090 | Protein of unknown function DUF3468 | | 11 |
| 70 | PEX2_065290 | hypothetical protein PEX2_065290 | | 11 |
| 71 | PEX2_065330 | Cerato-platanin | | 11 |
| 72 | PEX2_064160 | Cytochrome P450, E-class, group I | | 11 |
| 73 | PEX2_107230 | Pectin lyase fold/virulence factor | | 11 |
| 74 | PEX2_107240 | Amidase | | 11 |
| 75 | PEX2_107280 | Cytochrome P450 | | 11 |
| 76 | PEX2_107520 | hypothetical protein PEX2_107520 | | 11 |
| 77 | PEX2_107710 | Glycosyl transferase, family 15 | | 11 |
| 78 | PEX2_108150 | Glucose-methanol-choline oxidoreductase, C-terminal | | 11 |
| 79 | PEX2_108250 | FAD linked oxidase, N-terminal | | 11 |
| 80 | PEX2_108390 | Carboxylesterase, type B | | 11 |

| | | | | |
|---|---|---|---|---|
| 81 | PEX2_108490 | Protein disulfide isomerase | | 11 |
| 82 | PEX2_108900 | Glycoside hydrolase family 3 | | 11 |
| 83 | PEX2_109040 | FAD linked oxidase, N-terminal | | 11 |
| 84 | PEX2_109160 | Cytochrome P450 | | 11 |
| 85 | PEX2_109270 | Galactose oxidase/kelch, beta-propeller | | 11 |
| 86 | PEX2_109690 | Carboxylesterase, type B | | 11 |
| 87 | PEX2_109830 | Cytochrome P450 | | 11 |
| 88 | PEX2_110470 | Glycoside hydrolase, family 28 | | 11 |
| 89 | PEX2_110700 | Peptidase M35, deuterolysin | | 11 |
| 90 | PEX2_048450 | FAD linked oxidase, N-terminal | | 11 |
| 91 | PEX2_048600 | Glycoside hydrolase, family 28 | | 11 |
| 92 | PEX2_048660 | Lipase, class 3 | | 11 |
| 93 | PEX2_048700 | Six-hairpin glycosidase | | 11 |
| 94 | PEX2_048820 | O-methyltransferase, family 2 | | 11 |
| 95 | PEX2_082100 | Cytochrome P450 | | 11 |
| 96 | PEX2_082420 | Beta-lactamase-like protein | | 11 |
| 97 | PEX2_082440 | Glucose-methanol-choline oxidoreductase | | 11 |
| 98 | PEX2_082490 | Small GTPase superfamily, ARF/SAR type | | 11 |
| 99 | PEX2_082740 | Cytochrome P450 | | 11 |
| 100 | PEX2_082800 | Carboxylesterase, type B | | 11 |
| 101 | PEX2_082860 | Cytochrome P450 | | 11 |
| 102 | PEX2_083910 | Glucose-methanol-choline oxidoreductase, C-terminal | | 11 |
| 103 | PEX2_083030 | Multicopper oxidase, type 1 | | 11 |
| 104 | PEX2_083520 | Cytochrome P450 | | 11 |
| 105 | PEX2_048230 | Glucose-methanol-choline oxidoreductase | | 11 |
| 106 | PEX2_062350 | Carboxylesterase, type B | | 11 |
| 107 | PEX2_056700 | Cytochrome P450, E-class, CYP52 | | 11 |
| 108 | PEX2_056710 | Glycoside hydrolase, superfamily | | 11 |
| 109 | PEX2_057250 | hypothetical protein PEX2_057250 | | 11 |
| 110 | PEX2_057360 | Tyrosinase | | 11 |
| 111 | PEX2_059180 | Short-chain dehydrogenase/reductase SDR | | 11 |
| 112 | PEX2_059230 | Glycoside hydrolase, family 12 | | 11 |
| 113 | PEX2_059260 | Multicopper oxidase, type 2 | | 11 |
| 114 | PEX2_059890 | Glycoside hydrolase, superfamily | | 11 |
| 115 | PEX2_059920 | Glycoside hydrolase, family 61 | | 11 |
| 116 | PEX2_060980 | Peptidoglycan-binding Lysin subgroup | | 11 |
| 117 | PEX2_061300 | Glycoside hydrolase, family 13 | | 11 |
| 118 | PEX2_062680 | Cutinase | | 11 |
| 119 | PEX2_062950 | Carboxylesterase, type B | | 11 |
| 120 | PEX2_062960 | Carboxylesterase, type B | | 11 |
| 121 | PEX2_022780 | FAD linked oxidase, N-terminal | | 11 |
| 122 | PEX2_022990 | Carboxylesterase, type B | | 11 |
| 123 | PEX2_023310 | Glycoside hydrolase, superfamily | | 11 |
| 124 | PEX2_023630 | FAD linked oxidase, N-terminal | | 11 |

| | | | | |
|---|---|---|---|---|
| 125 | PEX2_023690 | Carboxylesterase, type B | | 11 |
| 126 | PEX2_023700 | Carboxylesterase, type B | | 11 |
| 127 | PEX2_024790 | Protein phosphatase 2C | | 11 |
| 128 | PEX2_024810 | Glycosyl transferase, family 1 | | 11 |
| 129 | PEX2_025880 | Alpha-mannosyltransferase | | 11 |
| 130 | PEX2_037880 | Glycoside hydrolase, superfamily | | 11 |
| 131 | PEX2_038970 | Autophagy-related protein 27 | | 11 |
| 132 | PEX2_039050 | Amidase | | 11 |
| 133 | PEX2_039220 | Multicopper oxidase, type 2 | | 11 |
| 134 | PEX2_039690 | Lipopolysaccharide-modifying protein | | 11 |
| 135 | PEX2_039710 | Phosphoesterase | | 11 |
| 136 | PEX2_038010 | Beta-hexosaminidase subunit alpha/beta | | 11 |
| 137 | PEX2_038230 | Glucose-methanol-choline oxidoreductase | | 11 |
| 138 | PEX2_070420 | Glycoside hydrolase, superfamily | | 11 |
| 139 | PEX2_072090 | Glycoside hydrolase, family 12 | | 11 |
| 140 | PEX2_090200 | hypothetical protein PEX2_090200 | | 11 |
| 141 | PEX2_090300 | Cytochrome P450 | | 11 |
| 142 | PEX2_090990 | Zinc finger, C2H2 | | 11 |
| 143 | PEX2_089270 | FAD linked oxidase, N-terminal | | 11 |
| 144 | PEX2_089980 | hypothetical protein PEX2_089980 | | 11 |
| 145 | PEX2_089670 | hypothetical protein PEX2_089670 | | 11 |
| 146 | PEX2_086510 | Glycoside hydrolase, superfamily | | 11 |
| 147 | PEX2_029630 | hypothetical protein PEX2_029630 | | 11 |
| 148 | PEX2_029950 | hypothetical protein PEX2_029950 | | 11 |
| 149 | PEX2_030000 | Bicupin, oxalate decarboxylase/oxidase | | 11 |
| 150 | PEX2_029040 | Cytochrome P450 | | 11 |
| 151 | PEX2_028700 | Glycoside hydrolase, family 7 | | 11 |
| 152 | PEX2_028710 | Glycoside hydrolase, superfamily | | 11 |
| 153 | PEX2_030560 | Phosphoesterase | | 11 |
| 154 | PEX2_030640 | Pectinesterase, catalytic | | 11 |
| 155 | PEX2_030850 | Glycoside hydrolase family 3 | | 11 |
| 156 | PEX2_049890 | Glycoside hydrolase/deacetylase, beta/alpha-barrel | | 11 |
| 157 | PEX2_049940 | Glycoside hydrolase, superfamily | | 11 |
| 158 | PEX2_049960 | FAD linked oxidase, N-terminal | | 11 |
| 159 | PEX2_005970 | hypothetical protein PEX2_005970 | | 11 |
| 160 | PEX2_006100 | Peptidase aspartic, catalytic | | 11 |
| 161 | PEX2_006340 | Mannose-binding lectin | | 11 |
| 162 | PEX2_077920 | UDP-glucose:Glycoprotein Glucosyltransferase | | 11 |
| 163 | PEX2_078650 | Phosphoesterase | | 11 |
| 164 | PEX2_078770 | Tyrosinase | | 11 |
| 165 | PEX2_079170 | Phosphoesterase | | 11 |
| 166 | PEX2_079180 | hypothetical protein PEX2_079180 | | 11 |
| 167 | PEX2_080070 | Monooxygenase, FAD-binding | | 11 |
| 168 | PEX2_080210 | Glycoside hydrolase, family 61 | | 11 |
| 169 | PEX2_097030 | Multicopper oxidase, type 2 | | 11 |

| | | | | |
|---|---|---|---|---|
| 170 | PEX2_097230 | Alpha-mannosyltransferase | | 11 |
| 171 | PEX2_097300 | Aldolase-type TIM barrel | | 11 |
| 172 | PEX2_075690 | Multicopper oxidase, type 3 | | 11 |
| 173 | PEX2_076170 | PIK-related kinase, FATC | | 11 |
| 174 | PEX2_098230 | Glycosyl transferase, family 15 | | 11 |
| 175 | PEX2_098310 | Chorismate mutase | | 11 |
| 176 | PEX2_076650 | Galactose oxidase/kelch, beta-propeller | | 11 |
| 177 | PEX2_076710 | Heat shock protein 70 family | | 11 |
| 178 | PEX2_076830 | Glycoside hydrolase, family 35 | | 11 |
| 179 | PEX2_076920 | hypothetical protein PEX2_076920 | | 11 |
| 180 | PEX2_076420 | putative domain, di-copper centre | | 11 |
| 181 | PEX2_097510 | Glycoside hydrolase, family 31 | | 11 |
| 182 | PEX2_097710 | Glycoside hydrolase/deacetylase, beta/alpha-barrel | | 11 |
| 183 | PEX2_004230 | Glycoside hydrolase family 3 | | 11 |
| 184 | PEX2_004440 | Heat shock protein 70 family | | 11 |
| 185 | PEX2_004690 | Lipase, secreted | | 11 |
| 186 | PEX2_004780 | Protein of unknown function DUF3468 | | 11 |
| 187 | PEX2_004840 | FAD linked oxidase, N-terminal | | 11 |
| 188 | PEX2_001850 | FAD linked oxidase, N-terminal | | 11 |
| 189 | PEX2_001930 | Cytochrome P450 | | 11 |
| 190 | PEX2_000290 | Multicopper oxidase, type 2 | | 11 |
| 191 | PEX2_000340 | Cytochrome P450 | | 11 |
| 192 | PEX2_088440 | FAD linked oxidase, N-terminal | | 11 |
| 193 | PEX2_088460 | Cytochrome P450 | | 11 |
| 194 | PEX2_020370 | FAD linked oxidase, N-terminal | | 11 |
| 195 | PEX2_020570 | Peptidoglycan-binding Lysin subgroup | | 11 |
| 196 | PEX2_021960 | Cytochrome P450, E-class, group I | | 11 |
| 197 | PEX2_022560 | hypothetical protein PEX2_022560 | | 11 |
| 198 | PEX2_040260 | Cytochrome P450 | | 11 |
| 199 | PEX2_040500 | Glycoside hydrolase, superfamily | | 11 |
| 200 | PEX2_040830 | Pectinesterase, catalytic | | 11 |
| 201 | PEX2_040900 | Antifungal protein | | 11 |
| 202 | PEX2_067240 | Glycoside hydrolase, superfamily | | 11 |
| 203 | PEX2_067280 | hypothetical protein PEX2_067280 | | 11 |
| 204 | PEX2_067310 | Bicupin, oxalate decarboxylase/oxidase | | 11 |
| 205 | PEX2_067580 | hypothetical protein PEX2_067580 | | 11 |
| 206 | PEX2_067610 | FAD linked oxidase, N-terminal | | 11 |
| 207 | PEX2_068400 | Cytochrome P450, E-class, group I | | 11 |
| 208 | PEX2_068850 | Glucose-methanol-choline oxidoreductase, N-terminal | | 11 |
| 209 | PEX2_069250 | Palmitoyl protein thioesterase | | 11 |
| 210 | PEX2_069460 | Carboxylesterase, type B | | 11 |
| 211 | PEX2_069880 | Glycoside hydrolase, family 28 | | 11 |
| 212 | PEX2_070070 | Transcription factor, fungi | | 11 |
| 213 | PEX2_065700 | Phosphoesterase | | 11 |

| | | | | |
|---|---|---|---|---|
| 214 | PEX2_091440 | Peptidoglycan-binding Lysin subgroup | | 11 |
| 215 | PEX2_093030 | Fungal chitosanase | | 11 |
| 216 | PEX2_073750 | Glycoside hydrolase, superfamily | | 11 |
| 217 | PEX2_072610 | hypothetical protein PEX2_072610 | | 11 |
| 218 | PEX2_072630 | FAD linked oxidase, N-terminal | | 11 |
| 219 | PEX2_072780 | FAD linked oxidase, N-terminal | | 11 |
| 220 | PEX2_072790 | Multicopper oxidase, type 3 | | 11 |
| 221 | PEX2_074260 | Carbon-nitrogen hydrolase | | 11 |
| 222 | PEX2_074360 | Amine oxidase | | 11 |
| 223 | PEX2_074610 | Flavin amine oxidase | | 11 |
| 224 | PEX2_074660 | Mitochondrial substrate/solute carrier | | 11 |
| 225 | PEX2_074910 | FAD linked oxidase, N-terminal | | 11 |
| 226 | PEX2_075090 | FAD linked oxidase, N-terminal | | 11 |
| 227 | PEX2_055090 | Cytochrome P450 | | 11 |
| 228 | PEX2_053300 | Glycoside hydrolase, family 13 | | 11 |
| 229 | PEX2_053990 | Glycoside hydrolase, superfamily | | 11 |
| 230 | PEX2_054130 | Glycoside hydrolase, family 61 | | 11 |
| 231 | PEX2_054250 | Glycoside hydrolase, superfamily | | 11 |
| 232 | PEX2_054440 | Carboxylesterase, type B | | 11 |
| 233 | PEX2_054830 | Sialidase family | | 11 |
| 234 | PEX2_052860 | Transcription factor, fungi | | 11 |
| 235 | PEX2_052910 | Carboxylesterase, type B | | 11 |
| 236 | PEX2_036290 | Flavin amine oxidase | | 11 |
| 237 | PEX2_036500 | Carboxylesterase, type B | | 11 |
| 238 | PEX2_035970 | Glycoside hydrolase family 3 | | 11 |
| 239 | PEX2_032670 | FAD linked oxidase, N-terminal | | 11 |
| 240 | PEX2_033310 | Peptidoglycan-binding Lysin subgroup | | 11 |
| 241 | PEX2_086800 | Endoplasmic reticulum, protein ERp29, C-terminal | | 11 |
| 242 | PEX2_086890 | hypothetical protein PEX2_086890 | | 11 |
| 243 | PEX2_087430 | Uncharacterized protein PEX2_087430 | | 11 |
| 244 | PEX2_087470 | Lipase, class 3 | | 11 |
| 245 | PEX2_087640 | FAD linked oxidase, N-terminal | | 11 |
| 246 | PEX2_087670 | Cytochrome P450, E-class, group I | | 11 |
| 247 | PEX2_100140 | Glycoside hydrolase, family 2, N-terminal | | 11 |
| 248 | PEX2_100710 | Histidine phosphatase superfamily, clade-1 | | 11 |
| 249 | PEX2_100760 | Transcription factor, fungi | | 11 |
| 250 | PEX2_099870 | Glycoside hydrolase, family 2, N-terminal | | 11 |
| 251 | PEX2_099890 | Glycoside hydrolase, superfamily | | 11 |
| 252 | PEX2_101550 | Carboxylesterase, type B | | 11 |
| 253 | PEX2_102040 | Cytochrome P450 | | 11 |
| 254 | PEX2_102630 | Short-chain dehydrogenase/reductase SDR | | 11 |
| 255 | PEX2_081580 | Histidine phosphatase superfamily, clade-2 | | 11 |
| 256 | PEX2_081710 | Fungal chitosanase | | 11 |
| 257 | PEX2_081830 | FAD linked oxidase, N-terminal | | 11 |
| 258 | PEX2_085130 | Glucose-methanol-choline oxidoreductase | | 11 |

| | | | | |
|---|---|---|---|---|
| 259 | PEX2_085850 | Cytochrome P450, E-class, group I | | 11 |
| 260 | PEX2_015750 | BIG/ATPase V1 complex, subunit S1 | | 11 |
| 261 | PEX2_015960 | Carboxylesterase, type B | | 11 |
| 262 | PEX2_016360 | Beta-hexosaminidase subunit alpha/beta | | 11 |
| 263 | PEX2_016600 | Glycoside hydrolase, family 71 | | 11 |
| 264 | PEX2_015270 | Peptidase aspartic, catalytic | | 11 |
| 265 | PEX2_015460 | N-acetylglucosaminyl phosphatidylinositol deacetylase | | 11 |
| 266 | PEX2_014310 | Chloroperoxidase | | 11 |
| 267 | PEX2_015040 | Phosphoesterase | | 11 |
| 268 | PEX2_013490 | Glycoside hydrolase, family 28 | | 11 |
| 269 | PEX2_013010 | Aromatic-ring-hydroxylating dioxygenase, alpha subunit | | 11 |
| 270 | PEX2_013180 | Glycoside hydrolase, family 28 | | 11 |
| 271 | PEX2_013190 | Pectinesterase, catalytic | | 11 |
| 272 | PEX2_013350 | FAD linked oxidase, N-terminal | | 11 |
| 273 | PEX2_012680 | Glycoside hydrolase, family 35 | | 11 |
| 274 | PEX2_012790 | hypothetical protein PEX2_012790 | | 11 |
| 275 | PEX2_019170 | Esterase, SGNH hydrolase-type | | 11 |
| 276 | PEX2_017600 | FAD-dependent pyridine nucleotide-disulfide oxidoreductase | | 11 |
| 277 | PEX2_017860 | KEN domain, ribonuclease activator | | 11 |
| 278 | PEX2_018010 | hypothetical protein PEX2_018010 | | 11 |
| 279 | PEX2_018720 | hypothetical protein PEX2_018720 | | 11 |
| 280 | PEX2_018940 | Protein of unknown function DUF3419 | | 11 |
| 281 | PEX2_019000 | Lipase, class 3 | | 11 |
| 282 | PEX2_046030 | Phosphoesterase | | 11 |
| 283 | PEX2_046210 | Heat shock protein DnaJ, N-terminal | | 11 |
| 284 | PEX2_046400 | Cytochrome P450 | | 11 |
| 285 | PEX2_046700 | Carboxylesterase, type B | | 11 |
| 286 | PEX2_047260 | hypothetical protein PEX2_047260 | | 11 |
| 287 | PEX2_047370 | Concanavalin A-like lectin/glucanases superfamily | | 11 |
| 288 | PEX2_047400 | Thioredoxin | | 11 |
| 289 | PEX2_047510 | Nuclease (SNase-like), OB-fold | | 11 |
| 290 | PEX2_043650 | Glycoside hydrolase, superfamily | | 11 |
| 291 | PEX2_044420 | Carboxylesterase, type B | | 11 |
| 292 | PEX2_044550 | hypothetical protein PEX2_044550 | | 11 |
| 293 | PEX2_042290 | Flavin amine oxidase | | 11 |
| 294 | PEX2_042520 | Cytochrome P450 | | 11 |
| 295 | PEX2_042850 | Glycoside hydrolase, family 31 | | 11 |
| 296 | PEX2_042980 | hypothetical protein PEX2_042980 | | 11 |
| 297 | PEX2_042150 | Antifungal protein | | 11 |
| 298 | PEX2_010780 | Carboxylesterase, type B | | 11 |
| 299 | PEX2_011360 | Glycoside hydrolase, superfamily | | 11 |
| 300 | PEX2_011410 | FAD linked oxidase, N-terminal | | 11 |

| | | | | |
|---|---|---|---|---|
| 301 | PEX2_011450 | Glycoside hydrolase/deacetylase, beta/alpha-barrel | | 11 |
| 302 | PEX2_044150 | Glucose-methanol-choline oxidoreductase | | 11 |

**Table S2.** List of significant GO terms associated with the number of DEGs for each sample comparison[1]

| R19 Apple vs. Conidia DEGs | | | |
|---|---|---|---|
| **GO Term** | **Number of DEGs** | **GO category** | **Richness factor** |
| catalytic activity | 1068 | MF | 0.23 |
| oxidoreductase activity | 689 | MF | 0.48 |
| hydrolase activity | 296 | MF | 0.18 |
| transmembrane transporter activity | 285 | MF | 0.31 |
| DNA binding | 277 | MF | 0.31 |
| zinc ion binding | 270 | MF | 0.36 |
| DNA-binding transcription factor activity, RNA polymerase II-specific | 203 | MF | 0.41 |
| monooxygenase activity | 143 | MF | 0.57 |
| oxidoreductase activity, acting on paired donors, with incorporation or reduction of molecular oxygen | 121 | MF | 0.54 |
| iron ion binding | 107 | MF | 0.51 |
| heme binding | 106 | MF | 0.53 |
| hydrolase activity, acting on glycosyl bonds | 77 | MF | 0.31 |
| flavin adenine dinucleotide binding | 54 | MF | 0.23 |
| methyltransferase activity | 49 | MF | 0.23 |
| oxidoreductase activity, acting on CH-OH group of donors | 37 | MF | 0.21 |
| carbohydrate binding | 29 | MF | 0.30 |
| phosphopantetheine binding | 25 | MF | 0.36 |
| hydrolase activity, acting on carbon-nitrogen (but not peptide) bonds | 23 | MF | 0.19 |
| NADP binding | 21 | MF | 0.32 |
| pyridoxal phosphate binding | 19 | MF | 0.25 |
| 3-oxoacyl-[acyl-carrier-protein] synthase activity | 16 | MF | 0.39 |
| oxidoreductase activity, acting on the CH-CH group of donors | 15 | MF | 0.26 |
| N,N-dimethylaniline monooxygenase activity | 13 | MF | 0.38 |
| phosphorelay sensor kinase activity | 11 | MF | 0.61 |
| copper ion binding | 11 | MF | 0.35 |
| channel activity | 11 | MF | 0.38 |
| microtubule binding | 8 | MF | 0.47 |
| serine-type carboxypeptidase activity | 7 | MF | 0.58 |
| microtubule motor activity | 6 | MF | 0.50 |
| polygalacturonase activity | 6 | MF | 0.55 |
| symporter activity | 6 | MF | 0.86 |
| nitronate monooxygenase activity | 5 | MF | 0.71 |
| cellulose binding | 5 | MF | 0.50 |

| | | | |
|---|---|---|---|
| NADPH dehydrogenase activity | 4 | MF | 0.80 |
| catalase activity | 4 | MF | 0.44 |
| urea transmembrane transporter activity | 4 | MF | 1.00 |
| hydrolase activity, acting on acid halide bonds, in C-halide compounds | 4 | MF | 0.67 |
| proton-transporting ATP synthase activity, rotational mechanism | 4 | MF | 0.67 |
| protein-arginine deiminase activity | 3 | MF | 1.00 |
| proton-exporting ATPase activity | 3 | MF | 0.75 |
| cellulase activity | 3 | MF | 0.60 |
| choline monooxygenase activity | 3 | MF | 0.60 |
| oleoyl-[acyl-carrier-protein] hydrolase activity | 2 | MF | 1.00 |
| glyceraldehyde-3-phosphate dehydrogenase (NAD+) (phosphorylating) activity | 2 | MF | 1.00 |
| hydroxymethylglutaryl-CoA reductase (NADPH) activity | 2 | MF | 1.00 |
| hydroxymethylglutaryl-CoA synthase activity | 2 | MF | 1.00 |
| isocitrate dehydrogenase (NAD+) activity | 2 | MF | 1.00 |
| pyruvate dehydrogenase (acetyl-transferring) activity | 2 | MF | 1.00 |
| (3R)-hydroxymyristoyl-[acyl-carrier-protein] dehydratase activity | 2 | MF | 1.00 |
| 3-hydroxydecanoyl-[acyl-carrier-protein] dehydratase activity | 2 | MF | 1.00 |
| myristoyl-[acyl-carrier-protein] hydrolase activity | 2 | MF | 1.00 |
| palmitoyl-[acyl-carrier-protein] hydrolase activity | 2 | MF | 1.00 |
| enoyl-[acyl-carrier-protein] reductase activity | 2 | MF | 1.00 |
| DNA polymerase processivity factor activity | 2 | MF | 1.00 |
| 3-hydroxyoctanoyl-[acyl-carrier-protein] dehydratase activity | 2 | MF | 1.00 |
| cholestenol delta-isomerase activity | 2 | MF | 1.00 |
| integral component of membrane | 1317 | CC | 0.44 |
| Nucleus | 311 | CC | 0.22 |
| plasma membrane | 89 | CC | 0.34 |
| extracellular region | 80 | CC | 0.63 |
| endoplasmic reticulum membrane | 35 | CC | 0.23 |
| anchored component of membrane | 13 | CC | 0.41 |
| Golgi membrane | 12 | CC | 0.34 |
| cell wall | 8 | CC | 0.36 |
| MCM complex | 6 | CC | 1.00 |
| fatty acid synthase complex | 4 | CC | 0.67 |
| myosin complex | 4 | CC | 0.57 |
| vacuolar lumen | 3 | CC | 1.00 |
| viral envelope | 3 | CC | 0.75 |
| COPI vesicle coat | 3 | CC | 0.60 |

| | | | |
|---|---|---|---|
| proton-transporting ATP synthase complex, catalytic core F(1) | 3 | CC | 0.75 |
| condensin complex | 2 | CC | 1.00 |
| Ndc80 complex | 2 | CC | 1.00 |
| cytoplasmic vesicle lumen | 2 | CC | 1.00 |
| transmembrane transport | 515 | BP | 0.51 |
| cellular metabolic process | 378 | BP | 0.12 |
| regulation of transcription by RNA polymerase II | 209 | BP | 0.38 |
| carbohydrate metabolic process | 184 | BP | 0.49 |
| Phosphorylation | 102 | BP | 0.24 |
| Proteolysis | 56 | BP | 0.18 |
| cellular amino acid metabolic process | 53 | BP | 0.19 |
| Methylation | 49 | BP | 0.23 |
| lipid biosynthetic process | 37 | BP | 0.21 |
| carbohydrate transport | 31 | BP | 0.51 |
| cell cycle | 23 | BP | 0.17 |
| polysaccharide catabolic process | 22 | BP | 0.22 |
| cell division | 22 | BP | 0.29 |
| fatty acid biosynthetic process | 18 | BP | 0.33 |
| protein glycosylation | 16 | BP | 0.33 |
| amino acid transport | 15 | BP | 0.38 |
| cell wall organization | 14 | BP | 0.41 |
| isoprenoid biosynthetic process | 13 | BP | 0.36 |
| phosphorelay signal transduction system | 11 | BP | 0.50 |
| sterol metabolic process | 10 | BP | 0.53 |
| terpenoid biosynthetic process | 9 | BP | 0.39 |
| cellulose catabolic process | 9 | BP | 0.50 |
| DNA replication initiation | 8 | BP | 0.73 |
| microtubule-based movement | 8 | BP | 0.53 |
| ATP synthesis coupled proton transport | 8 | BP | 0.62 |
| sterol biosynthetic process | 8 | BP | 0.47 |
| DNA duplex unwinding | 8 | BP | 0.35 |
| acetyl-CoA metabolic process | 7 | BP | 0.64 |
| signal peptide processing | 4 | BP | 0.50 |
| phosphate ion transport | 4 | BP | 0.57 |
| oligopeptide transport | 4 | BP | 0.80 |
| reciprocal meiotic recombination | 4 | BP | 0.67 |
| protein-chromophore linkage | 4 | BP | 1.00 |
| hydrogen peroxide catabolic process | 4 | BP | 0.44 |
| urea transmembrane transport | 4 | BP | 1.00 |
| glycine catabolic process | 3 | BP | 1.00 |
| mitotic chromosome condensation | 3 | BP | 1.00 |
| putrescine biosynthetic process | 3 | BP | 1.00 |

| | | | |
|---|---|---|---|
| peroxisome fission | 3 | BP | 0.75 |
| glycine betaine biosynthetic process from choline | 3 | BP | 0.60 |
| carbohydrate transmembrane transport | 3 | BP | 1.00 |
| N-acetylglucosamine metabolic process | 2 | BP | 1.00 |
| acetyl-CoA biosynthetic process from pyruvate | 2 | BP | 1.00 |
| protein ADP-ribosylation | 2 | BP | 1.00 |
| osmosensory signaling pathway via Sho1 osmosensor | 2 | BP | 1.00 |
| peptidoglycan catabolic process | 2 | BP | 1.00 |
| detection of visible light | 2 | BP | 1.00 |
| farnesyl diphosphate biosynthetic process, mevalonate pathway | 2 | BP | 1.00 |
| melanin biosynthetic process | 2 | BP | 1.00 |
| intermembrane lipid transfer | 2 | BP | 1.00 |
| **RS1 Apple vs. Conidia DEGs** | | | |
| hydrolase activity | 226 | MF | 0.14 |
| transmembrane transporter activity | 178 | MF | 0.19 |
| DNA-binding transcription factor activity | 87 | MF | 0.16 |
| hydrolase activity, acting on ester bonds | 40 | MF | 0.12 |
| carbohydrate binding | 25 | MF | 0.26 |
| hydrolase activity, acting on carbon-nitrogen (but not peptide) bonds | 21 | MF | 0.18 |
| NADP binding | 15 | MF | 0.23 |
| motor activity | 12 | MF | 0.67 |
| polygalacturonase activity | 11 | MF | 1.00 |
| microtubule binding | 11 | MF | 0.65 |
| N,N-dimethylaniline monooxygenase activity | 10 | MF | 0.29 |
| microtubule motor activity | 9 | MF | 0.75 |
| carboxypeptidase activity | 9 | MF | 0.35 |
| cellulose binding | 8 | MF | 0.80 |
| phosphorelay sensor kinase activity | 7 | MF | 0.39 |
| DNA helicase activity | 7 | MF | 0.30 |
| 2 iron, 2 sulfur cluster binding | 7 | MF | 0.28 |
| sulfuric ester hydrolase activity | 6 | MF | 0.43 |
| serine-type carboxypeptidase activity | 4 | MF | 0.33 |
| MAP kinase activity | 4 | MF | 0.67 |
| voltage-gated ion channel activity | 4 | MF | 0.57 |
| mannan endo-1,6-alpha-mannosidase activity | 4 | MF | 0.50 |
| metalloaminopeptidase activity | 4 | MF | 0.36 |
| dolichyl-phosphate-mannose-protein mannosyltransferase activity | 3 | MF | 1.00 |
| chloride channel activity | 3 | MF | 0.60 |
| calcium:proton antiporter activity | 3 | MF | 0.43 |
| choline monooxygenase activity | 3 | MF | 0.60 |

| | | | |
|---|---|---|---|
| manganese ion binding | 3 | MF | 0.43 |
| tRNA-intron endonuclease activity | 2 | MF | 1.00 |
| protein disulfide isomerase activity | 2 | MF | 0.67 |
| 5-methyltetrahydropteroyltriglutamate-homocysteine S-methyltransferase activity | 2 | MF | 0.67 |
| succinate-semialdehyde dehydrogenase (NAD+) activity | 2 | MF | 0.67 |
| mating-type factor pheromone receptor activity | 2 | MF | 1.00 |
| osmosensor activity | 2 | MF | 0.67 |
| 4-alpha-hydroxytetrahydrobiopterin dehydratase activity | 2 | MF | 0.67 |
| ammonium transmembrane transporter activity | 2 | MF | 0.67 |
| pyrimidine nucleotide-sugar transmembrane transporter activity | 2 | MF | 1.00 |
| P-P-bond-hydrolysis-driven protein transporting | 2 | MF | 0.67 |
| DNA polymerase processivity factor activity | 2 | MF | 1.00 |
| succinate-semialdehyde dehydrogenase (NADP+) activity | 2 | MF | 0.67 |
| glutathione hydrolase activity | 2 | MF | 1.00 |
| cholestenol delta-isomerase activity | 2 | MF | 1.00 |
| sulfur dioxygenase activity | 2 | MF | 1.00 |
| 2-(3-amino-3-carboxypropyl)histidine synthase activity | 2 | MF | 1.00 |
| hypoglycin A gamma-glutamyl transpeptidase activity | 2 | MF | 1.00 |
| leukotriene C4 gamma-glutamyl transferase activity | 2 | MF | 1.00 |
| integral component of membrane | 837 | CC | 0.28 |
| plasma membrane | 47 | CC | 0.18 |
| extracellular region | 31 | CC | 0.24 |
| Cytoskeleton | 31 | CC | 0.31 |
| endoplasmic reticulum membrane | 29 | CC | 0.19 |
| Nucleolus | 27 | CC | 0.36 |
| Microtubule | 16 | CC | 0.33 |
| Golgi membrane | 14 | CC | 0.40 |
| anchored component of membrane | 11 | CC | 0.34 |
| cell wall | 6 | CC | 0.27 |
| small-subunit processome | 6 | CC | 0.50 |
| COPI vesicle coat | 5 | CC | 1.00 |
| MCM complex | 5 | CC | 0.83 |
| Nucleosome | 4 | CC | 0.40 |
| integral component of plasma membrane | 4 | CC | 0.40 |
| myosin complex | 4 | CC | 0.57 |
| outer membrane | 4 | CC | 0.19 |
| DASH complex | 4 | CC | 0.40 |
| sno(s)RNA-containing ribonucleoprotein complex | 3 | CC | 0.50 |
| dynein complex | 3 | CC | 0.75 |
| tRNA-intron endonuclease complex | 2 | CC | 0.67 |
| signal peptidase complex | 2 | CC | 0.67 |

| | | | |
|---|---|---|---|
| endoplasmic reticulum lumen | 2 | CC | 0.67 |
| COPII vesicle coat | 2 | CC | 0.67 |
| Ndc80 complex | 2 | CC | 1.00 |
| transmembrane transport | 329 | BP | 0.33 |
| carbohydrate metabolic process | 140 | BP | 0.37 |
| Proteolysis | 40 | BP | 0.13 |
| ribosome biogenesis | 38 | BP | 0.37 |
| Methylation | 36 | BP | 0.17 |
| cell division | 27 | BP | 0.35 |
| rRNA processing | 26 | BP | 0.38 |
| cell cycle | 24 | BP | 0.18 |
| amino acid transport | 19 | BP | 0.49 |
| microtubule-based process | 17 | BP | 0.47 |
| carbohydrate transport | 17 | BP | 0.28 |
| protein glycosylation | 16 | BP | 0.33 |
| cation transmembrane transport | 14 | BP | 0.12 |
| cytoskeleton organization | 13 | BP | 0.27 |
| cell wall organization | 13 | BP | 0.38 |
| microtubule-based movement | 11 | BP | 0.73 |
| Mannosylation | 10 | BP | 0.38 |
| phosphorelay signal transduction system | 8 | BP | 0.36 |
| DNA duplex unwinding | 7 | BP | 0.30 |
| calcium ion transmembrane transport | 7 | BP | 0.35 |
| DNA replication initiation | 6 | BP | 0.55 |
| iron-sulfur cluster assembly | 6 | BP | 0.43 |
| acyl-CoA metabolic process | 4 | BP | 0.27 |
| sterol metabolic process | 4 | BP | 0.21 |
| peptidyl-diphthamide biosynthetic process from peptidyl-histidine | 4 | BP | 0.80 |
| glycogen biosynthetic process | 3 | BP | 0.75 |
| trehalose biosynthetic process | 3 | BP | 0.60 |
| chitin biosynthetic process | 3 | BP | 0.43 |
| potassium ion transport | 3 | BP | 0.50 |
| retrograde vesicle-mediated transport, Golgi to endoplasmic reticulum | 3 | BP | 0.43 |
| reciprocal meiotic recombination | 3 | BP | 0.50 |
| glycine betaine biosynthetic process from choline | 3 | BP | 0.60 |
| protein O-linked mannosylation | 3 | BP | 1.00 |
| xylan catabolic process | 3 | BP | 0.43 |
| tetrahydrofolate biosynthetic process | 3 | BP | 0.60 |
| cleavage involved in rRNA processing | 2 | BP | 0.67 |
| N-acetylglucosamine metabolic process | 2 | BP | 1.00 |
| DNA topological change | 2 | BP | 0.67 |

| | | | |
|---|---|---|---|
| DNA catabolic process | 2 | BP | 1.00 |
| tetrahydrobiopterin biosynthetic process | 2 | BP | 0.67 |
| osmosensory signaling pathway via Sho1 osmosensor | 2 | BP | 1.00 |
| peptidyl-lysine modification to peptidyl-hypusine | 2 | BP | 1.00 |
| putrescine biosynthetic process | 2 | BP | 0.67 |
| gamma-aminobutyric acid catabolic process | 2 | BP | 0.67 |
| siderophore biosynthetic process | 2 | BP | 0.67 |
| maintenance of protein location in cell cortex | 2 | BP | 0.67 |
| plasma membrane fusion involved in cytogamy | 2 | BP | 1.00 |
| folic acid biosynthetic process | 2 | BP | 1.00 |
| stress-activated MAPK cascade | 2 | BP | 1.00 |
| ammonium transmembrane transport | 2 | BP | 0.67 |
| pyrimidine nucleotide-sugar transmembrane transport | 2 | BP | 1.00 |
| intermembrane lipid transfer | 2 | BP | 1.00 |
| **R19 vs. RS1 Apple DEGs** | | | |
| catalytic activity | 634 | MF | 0.14 |
| oxidoreductase activity | 375 | MF | 0.26 |
| hydrolase activity | 224 | MF | 0.14 |
| transferase activity | 181 | MF | 0.13 |
| DNA binding | 163 | MF | 0.18 |
| zinc ion binding | 156 | MF | 0.21 |
| DNA-binding transcription factor activity, RNA polymerase II-specific | 123 | MF | 0.25 |
| transmembrane transporter activity | 102 | MF | 0.11 |
| monooxygenase activity | 50 | MF | 0.20 |
| oxidoreductase activity, acting on paired donors, with incorporation or reduction of molecular oxygen | 42 | MF | 0.19 |
| iron ion binding | 39 | MF | 0.19 |
| heme binding | 39 | MF | 0.20 |
| phosphopantetheine binding | 28 | MF | 0.40 |
| FAD binding | 25 | MF | 0.18 |
| 3-oxoacyl-[acyl-carrier-protein] synthase activity | 22 | MF | 0.54 |
| ATPase-coupled transmembrane transporter activity | 21 | MF | 0.26 |
| N-acetyltransferase activity | 20 | MF | 0.24 |
| O-methyltransferase activity | 16 | MF | 0.52 |
| dioxygenase activity | 16 | MF | 0.11 |
| serine-type peptidase activity | 15 | MF | 0.25 |
| FMN binding | 11 | MF | 0.24 |
| carbohydrate binding | 11 | MF | 0.11 |
| ADP binding | 10 | MF | 0.48 |
| NADP binding | 9 | MF | 0.14 |
| aspartic-type endopeptidase activity | 8 | MF | 0.33 |
| serine-type endopeptidase activity | 8 | MF | 0.26 |

| | | | |
|---|---|---|---|
| chitin binding | 7 | MF | 0.27 |
| metalloendopeptidase activity | 4 | MF | 0.19 |
| structural constituent of cell wall | 4 | MF | 0.80 |
| tetrahydrofolylpolyglutamate synthase activity | 3 | MF | 0.60 |
| glutathione transferase activity | 3 | MF | 0.38 |
| beta-galactosidase activity | 3 | MF | 0.43 |
| calcium:proton antiporter activity | 3 | MF | 0.43 |
| NADPH dehydrogenase activity | 2 | MF | 0.40 |
| alpha-amylase activity | 2 | MF | 0.50 |
| oxidoreductase activity, acting on NAD(P)H, NAD(P) as acceptor | 2 | MF | 0.67 |
| choline monooxygenase activity | 2 | MF | 0.40 |
| nitrate reductase (NADPH) activity | 2 | MF | 0.67 |
| 6-methylsalicylic acid synthase activity | 2 | MF | 1.00 |
| alpha-amylase activity (releasing maltohexaose) | 2 | MF | 0.50 |
| starch binding | 2 | MF | 0.67 |
| integral component of membrane | 590 | CC | 0.20 |
| Nucleus | 166 | CC | 0.12 |
| extracellular region | 36 | CC | 0.28 |
| plasma membrane | 28 | CC | 0.11 |
| kinesin complex | 6 | CC | 0.46 |
| fungal-type cell wall | 4 | CC | 1.00 |
| vacuolar lumen | 2 | CC | 0.67 |
| beta-galactosidase complex | 2 | CC | 0.67 |
| cytoplasmic vesicle lumen | 2 | CC | 1.00 |
| regulation of transcription by RNA polymerase II | 123 | BP | 0.22 |
| transmembrane transport | 104 | BP | 0.10 |
| Proteolysis | 38 | BP | 0.12 |
| carbohydrate metabolic process | 36 | BP | 0.10 |
| Methylation | 35 | BP | 0.17 |
| ion transport | 31 | BP | 0.06 |
| fatty acid biosynthetic process | 24 | BP | 0.44 |
| polysaccharide catabolic process | 19 | BP | 0.19 |
| nucleoside metabolic process | 17 | BP | 0.29 |
| glutathione metabolic process | 11 | BP | 0.24 |
| terpenoid biosynthetic process | 8 | BP | 0.35 |
| carbohydrate transport | 7 | BP | 0.11 |
| aromatic amino acid family metabolic process | 7 | BP | 0.15 |
| oligopeptide transport | 3 | BP | 0.60 |
| oxalate metabolic process | 3 | BP | 0.75 |
| nitrate assimilation | 3 | BP | 0.60 |
| xylan catabolic process | 3 | BP | 0.43 |
| tetrahydrofolylpolyglutamate biosynthetic process | 3 | BP | 0.60 |

| | | | |
|---|---|---|---|
| glycine betaine biosynthetic process from choline | 2 | BP | 0.40 |
| negative regulation of phosphoprotein phosphatase activity | 2 | BP | 0.67 |
| conidium formation | 2 | BP | 0.29 |
| **R19 vs. RS1 Conidia DEGs** | | | |
| catalytic activity | 1273 | MF | 0.27 |
| oxidoreductase activity | 491 | MF | 0.34 |
| transmembrane transporter activity | 446 | MF | 0.49 |
| hydrolase activity | 436 | MF | 0.26 |
| DNA binding | 322 | MF | 0.36 |
| zinc ion binding | 313 | MF | 0.41 |
| DNA-binding transcription factor activity, RNA polymerase II-specific | 226 | MF | 0.45 |
| nucleic acid binding | 130 | MF | 0.09 |
| monooxygenase activity | 97 | MF | 0.39 |
| oxidoreductase activity, acting on paired donors, with incorporation or reduction of molecular oxygen | 84 | MF | 0.38 |
| heme binding | 81 | MF | 0.41 |
| iron ion binding | 79 | MF | 0.38 |
| methyltransferase activity | 66 | MF | 0.31 |
| dioxygenase activity | 57 | MF | 0.40 |
| FAD binding | 48 | MF | 0.35 |
| phosphopantetheine binding | 42 | MF | 0.60 |
| flavin adenine dinucleotide binding | 35 | MF | 0.15 |
| oxidoreductase activity, acting on CH-OH group of donors | 33 | MF | 0.19 |
| 3-oxoacyl-[acyl-carrier-protein] synthase activity | 27 | MF | 0.66 |
| D-threo-aldose 1-dehydrogenase activity | 20 | MF | 0.42 |
| ADP binding | 17 | MF | 0.81 |
| beta-glucosidase activity | 13 | MF | 0.65 |
| O-methyltransferase activity | 13 | MF | 0.42 |
| scopolin beta-glucosidase activity | 12 | MF | 0.63 |
| RNA-DNA hybrid ribonuclease activity | 11 | MF | 0.55 |
| RNA helicase activity | 11 | MF | 0.37 |
| DNA-directed 5'-3' RNA polymerase activity | 10 | MF | 0.30 |
| N,N-dimethylaniline monooxygenase activity | 8 | MF | 0.24 |
| holo-[acyl-carrier-protein] synthase activity | 5 | MF | 0.83 |
| polygalacturonase activity | 5 | MF | 0.45 |
| cellulose binding | 4 | MF | 0.40 |
| hydrolase activity, acting on acid halide bonds | 4 | MF | 0.67 |
| alpha-amylase activity (releasing maltohexaose) | 3 | MF | 0.75 |
| urea transmembrane transporter activity | 3 | MF | 0.75 |
| proton-exporting ATPase activity | 3 | MF | 0.75 |
| inorganic phosphate transmembrane transporter activity | 3 | MF | 0.43 |
| alpha-amylase activity | 3 | MF | 0.75 |

| | | | |
|---|---|---|---|
| nitrate reductase (NADPH) activity | 2 | MF | 0.67 |
| UMP kinase activity | 2 | MF | 1.00 |
| S-formylglutathione hydrolase activity | 2 | MF | 1.00 |
| mannose-6-phosphate isomerase activity | 2 | MF | 0.67 |
| cytidylate kinase activity | 2 | MF | 1.00 |
| carbamoyl-phosphate synthase (glutamine-hydrolyzing) activity | 2 | MF | 1.00 |
| aminoacyl-tRNA hydrolase activity | 2 | MF | 1.00 |
| adenylylsulfate kinase activity | 2 | MF | 1.00 |
| D-arabinono-1,4-lactone oxidase activity | 2 | MF | 0.67 |
| integral component of membrane | 1207 | CC | 0.41 |
| Nucleus | 345 | CC | 0.24 |
| plasma membrane | 78 | CC | 0.30 |
| extracellular region | 67 | CC | 0.53 |
| Nucleolus | 45 | CC | 0.59 |
| mitochondrial membrane | 14 | CC | 0.08 |
| kinesin complex | 10 | CC | 0.77 |
| small-subunit processome | 6 | CC | 0.50 |
| MCM complex | 4 | CC | 0.67 |
| preribosome, large subunit precursor | 4 | CC | 0.80 |
| fatty acid synthase complex | 4 | CC | 0.67 |
| viral envelope | 3 | CC | 0.75 |
| RNA polymerase III complex | 3 | CC | 0.43 |
| transmembrane transport | 470 | BP | 0.47 |
| transcription, DNA-templated | 302 | BP | 0.38 |
| regulation of transcription, DNA-templated | 262 | BP | 0.40 |
| regulation of transcription by RNA polymerase II | 231 | BP | 0.42 |
| Methylation | 66 | BP | 0.32 |
| ion transport | 66 | BP | 0.12 |
| carbohydrate metabolic process | 66 | BP | 0.18 |
| ribosome biogenesis | 53 | BP | 0.51 |
| rRNA processing | 38 | BP | 0.55 |
| fatty acid biosynthetic process | 31 | BP | 0.56 |
| nucleoside metabolic process | 29 | BP | 0.50 |
| carbohydrate catabolic process | 25 | BP | 0.17 |
| lipid biosynthetic process | 25 | BP | 0.14 |
| carbohydrate transport | 23 | BP | 0.38 |
| terpenoid biosynthetic process | 16 | BP | 0.70 |
| alkaloid metabolic process | 14 | BP | 0.64 |
| amino acid transport | 13 | BP | 0.33 |
| ribosomal large subunit biogenesis | 11 | BP | 0.73 |
| cellulose catabolic process | 10 | BP | 0.56 |
| L-phenylalanine catabolic process | 6 | BP | 0.60 |

| | | | |
|---|---|---|---|
| lipid glycosylation | 5 | BP | 0.56 |
| protein phosphopantetheinylation | 5 | BP | 0.83 |
| DNA replication initiation | 5 | BP | 0.45 |
| maturation of LSU-rRNA | 5 | BP | 0.63 |
| ribosomal large subunit assembly | 5 | BP | 1.00 |
| nitrate assimilation | 4 | BP | 0.80 |
| de novo' pyrimidine nucleobase biosynthetic process | 4 | BP | 0.67 |
| proton export across plasma membrane | 3 | BP | 1.00 |
| urea transmembrane transport | 3 | BP | 0.75 |
| hydrogen sulfide biosynthetic process | 3 | BP | 1.00 |
| formaldehyde catabolic process | 3 | BP | 1.00 |
| aflatoxin biosynthetic process | 3 | BP | 0.75 |
| long-chain fatty acid biosynthetic process | 3 | BP | 0.75 |
| oxalate metabolic process | 3 | BP | 0.75 |
| removal of superoxide radicals | 3 | BP | 0.60 |
| lysine biosynthetic process via diaminopimelate | 3 | BP | 1.00 |
| trehalose biosynthetic process | 3 | BP | 0.60 |
| maturation of 5.8S rRNA from tricistronic rRNA transcript (SSU-rRNA, 5.8S rRNA, LSU-rRNA) | 3 | BP | 0.60 |
| maturation of LSU-rRNA from tricistronic rRNA transcript (SSU-rRNA, 5.8S rRNA, LSU-rRNA) | 3 | BP | 0.60 |
| sulfate assimilation | 3 | BP | 0.75 |
| mitochondrial phosphate ion transmembrane transport | 2 | BP | 0.67 |
| siderophore biosynthetic process | 2 | BP | 0.67 |
| peptidyl-lysine modification to peptidyl-hypusine | 2 | BP | 1.00 |

[1]Richness factor = number of DEGs in a category/ total number of genes in a genome annotated in that category. BP = biological processes, CC = cellular component, MF = molecular function
